# Multi- and All-Acceptor Polymers for High-Performance n-Type Polymer Field Effect Transistors

**DOI:** 10.3390/polym18010080

**Published:** 2025-12-27

**Authors:** Ganapathi Bharathi, Seongin Hong

**Affiliations:** 1 Department of Physics and Semiconductor Science, Gachon University, Seongnam 13120, Republic of Korea; bharathigm@gachon.ac.kr; 2 Department of Semiconductor Engineering, Gachon University, Seongnam 13120, Republic of Korea

**Keywords:** n-type polymers, multi-acceptor strategy, frontier molecular orbitals, electron mobility, polymer field effect transistors

## Abstract

Multi-acceptor and all-acceptor polymers solve the fundamental challenge of achieving unipolar electron transport without compromising stability in n-type polymer field-effect transistors. By systematically replacing electron-rich donors with acceptor units, these architectures push LUMO levels below −4.0 eV and HOMO levels below −5.7 eV. Consequently, electron mobilities exceeding 7 cm^2^ V^−1^ s^−1^, on/off ratios approaching 10^7^, and months-long ambient operation can be achieved. This review connects the molecular architecture to device function. We assert that short-range π-aggregation matters more than crystallinity—tight π-stacking over 5–10 molecules drives transport in rigid backbones. Device optimization through interface engineering (e.g., amine-functionalized self-assembled monolayers reduce the threshold voltages to 1–5 V), contact resistance minimization, and controlled processing transform the intrinsic material potential into working transistors. Current challenges, such as balancing the operating voltage against stability, scaling synthetic yields, and reducing contact resistance, define near-term research directions toward complementary circuits, thermoelectrics, and bioelectronics.

## 1. Introduction

Electron-transporting (n-type) polymer semiconductors are essential but underdeveloped components of organic electronics. Their importance stems from the fundamental architecture of modern electronic devices, such as complementary metal-oxide-semiconductor (CMOS) logic circuits, p–n junctions, and rectifying interfaces, all of which require balanced charge transport from both carrier types [1,2,3,4]. Without efficient n-type materials, organic electronics remain confined to simple unipolar p-type circuits, which consume excessive static power, lack signal amplification capabilities, and cannot realize sophisticated logic functions. Complementary circuits combining p- and n-type transistors offer significant advantages, such as voltage gains exceeding 44 V/V, noise margins approaching 70% of the theoretical limits, and operation at sub-volt supply voltages, which are critical performance metrics for wearable electronics, bioelectronic sensors, and distributed sensor networks [5]. In addition to digital logic, n-type polymers are essential for other applications such as polymer cells [6]. and organic thermoelectrics [7,8]. Moreover, organic electrochemical transistors require n-type channels for biosensing applications that require low-voltage aqueous operations [9]. The functional necessity is clear; realizing the full potential of organic electronics depends on closing the performance gap between p- and n-type polymer semiconductors. However, the disparity in performance remained significant.

P-type polymers have achieved hole mobilities exceeding 10 cm^2^ V^−1^ s^−1^ through decades of systematic molecular engineering, establishing well-understood design principles connecting backbone planarity, alkyl chain engineering, and charge transport efficiency [2,10]. The benchmark n-type polymer P(NDI2OD-T2) (widely known as N2200) demonstrates electron mobilities of up to 6 cm^2^ V^−1^ s^−1^ under optimal conditions, a decent value, but fundamentally limited by structural constraints inherent to conventional donor-acceptor (D–A) architectures [11]. Notably, careful optimization of the acceptor strength and molecular design within D–A frameworks has yielded higher performances; the strongly electron-deficient F_4_BDOPV-2T copolymer achieved apparent electron mobilities of 14.9 cm^2^ V^−1^ s^−1^ in air, surpassing prior n-type benchmarks by several-fold [12]. However, such exceptional results remain sporadic, motivating the exploration of alternative polymer architectures, particularly multi- and all-acceptor (A–A) designs, to systematically achieve higher transport efficiency and air stability. This fundamental limitation stems from the inherent electronic contradictions in the D–A polymers. The conventional n-type D–A polymers suffer from persistent ambipolar behavior, high off-currents (>10^−8^ A), modest on/off ratios (<10^5^), and substantial hole injection that compromises unipolar operation [4,13]. This electronic structure compromises the traces to the electron-rich donor units (typically bithiophene derivatives) that, despite facilitating synthetic tractability and solubility, inevitably elevate both the highest occupied molecular orbital (HOMO) and lowest unoccupied molecular orbital (LUMO) energy levels through intramolecular charge transfer (ICT). Even incorporating strong electron-withdrawing groups into acceptor moieties is insufficient because the donor segments persist in elevating the frontier orbital energies to ranges incompatible with unipolar n-type behavior.

Fundamental difficulties exist in the preparation of high-performance n-type polymers that operate at multiple levels. The origin traces to an unavoidable electronic paradox: electron-rich donor units, necessary for synthetic flexibility and solubility, inherently maintain shallow HOMO levels (approximately −5.0 to −5.2 eV) that permit facile hole injection despite concurrent acceptor-driven LUMO deepening. Thermodynamically, electrons in n-type polymers face hostile environments—atmospheric oxygen undergoes two- and four-electron reductions at potentials of approximately −4.4 and −4.9 eV versus vacuum, respectively, whereas water reduction potential lies near −3.7 eV [14].

Electrons injected into polymer LUMO levels transfer readily to physisorbed oxygen, forming superoxide radicals (O_2_^−^) that function as Coulombic scattering centers and electron traps. Water molecules adsorbed at semiconductor-dielectric interfaces create additional deep trap states through dipolar interactions with the accumulated charge. These ambient degradation pathways demand LUMO levels below −4.0 eV for kinetic stability—a threshold requiring exceptionally strong electron-withdrawing building blocks. Beyond stability concerns, synthetic accessibility presents severe constraints. The library of sufficiently electron-deficient monomers remains limited compared to their electron-rich counterparts developed for p-type systems. Strong acceptor units often incorporate multiple carbonyl or cyano groups that, while deepening frontier orbitals, simultaneously introduce steric congestion inducing backbone torsion—dihedral angles exceeding 20° dramatically reduce effective conjugation and π-orbital overlap [3]. Furthermore, the preparation of organometallic reagents (boronic esters and stannanes) from highly electron-deficient heterocycles encounters yield and stability problems that complicate polymerization chemistry [13].

Current strategies for addressing the limitations of n-type polymers span several approaches with varying degrees of success. The substitution of sp^2^carbon with sp^2^nitrogen, oxygen, or selenium in aromatic rings lowers the LUMO levels through electronegativity effects, while sometimes enabling favorable non-covalent conformational locks [13,15]. Halogenation, particularly fluorination, reduces frontier orbital energies and enhances ambient stability—fluorinated polymers like P6F-C3 achieve electron mobilities of 4.97 cm^2^ V^−1^ s^−1^, though often retain ambipolar characteristics [16]. Cyano-group incorporation provides powerful electron withdrawal, but disrupts backbone planarity if positioned improperly [13,17]. Extended conjugation through fused ring systems such as naphthalene diimide (NDI) or benzodifurandione-based oligo(p-phenylene vinylene) (BDOPV) creates large π-systems with deep LUMOs, exemplified by BDOPV derivatives showing both high electron mobility and efficient n-type doping for thermoelectrics [18]. These strategies have achieved notable successes but encounter a persistent limitation—modifications to conventional D–A architectures typically improve one parameter (mobility or stability) while compromising others, rarely achieving the simultaneous combination of high electron mobility, unipolar transport characteristics, low operating voltages, and ambient stability required for practical applications.

Multi-acceptor and A–A architectures address these challenges through the systematic elimination of electron-rich components. The strategy progresses logically: dual-acceptor systems (D–A_1_–D–A_2_ or A_1_–A_2_) reduce donor content, triple-acceptor configurations (A–D–A–D–A) minimize donor segments further, and A–A homopolymers eliminate them entirely [1,11]. Each architectural refinement deepens both the LUMO and HOMO levels; enhanced electron affinity from multiple acceptor units lowers the LUMO, whereas reduced ICT simultaneously depresses the HOMO. The electronic structure consequences prove dramatic: A–A polymers like poly(bithiazolothienyl-tetracarboxydiimide) achieve LUMO levels approaching −4.0 eV for ambient stability alongside HOMO levels below −5.5 eV that effectively suppress hole injection [4,11]. This deep HOMO positioning delivers the critical distinction from modified D–A polymers—off-currents dropping to 10^−10^–10^−11^ A ranges with on/off ratios exceeding 10^7^, true unipolar characteristics unattainable through incremental modifications of conventional architectures. The triple-acceptor polymer pDFB-TF exemplifies balanced optimization: electron mobility of 5.04 cm^2^ V^−1^ s^−1^ with unipolar transport, crystalline coherence length (CCL) of 524 Å, and π-π stacking distance of 3.62 Å—performance arising from fluorination-enabled conformational locking that simultaneously deepens frontier orbitals and rigidifies backbone geometry [2]. Critically, these polymers maintain performance over weeks in ambient conditions where benchmark N2200 degrades to 0.01 cm^2^ V^−1^ s^−1^ within 14 weeks, demonstrating that multi-acceptor design enables fundamentally different stability profiles [3].

**Figure 1:**
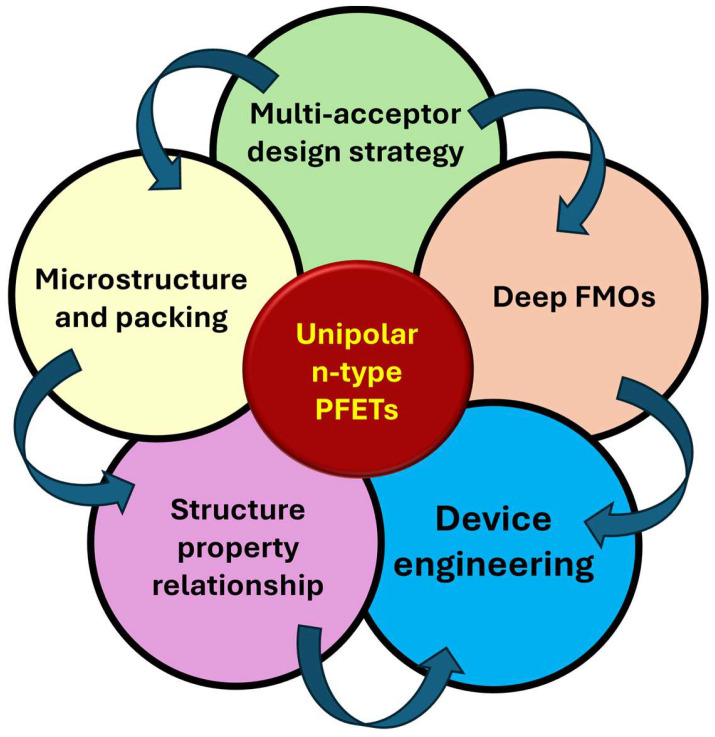
Schematic overview illustrating the key design and performance parameters governing high-performance unipolar n-type polymer field-effect transistors (PFETs). The central objective is supported by five interconnected aspects: (i) multi-acceptor design strategy for enhanced electron affinity, (ii) deep frontier molecular orbitals (FMOs) ensuring air stability, (iii) microstructure and molecular packing for efficient charge transport, (iv) structure–property relationships linking molecular architecture to device behavior, and (v) device engineering optimizing interfacial and contact properties.

As summarized in Figure 1, achieving high-performance unipolar n-type polymer field-effect transistors (PFETs) relies on a synergistic interplay between the molecular design (multi-acceptor frameworks and deep frontier molecular orbitals (FMOs)), microstructural organization, and device-level engineering, which collectively determine the charge transport efficiency and operational stability. This review systematically examines multi-acceptor and A–A polymer strategies for high-performance n-type transistors. Section 2 establishes the key metrics for evaluating performance: frontier orbital energies, mobility extraction, and contact resistance, which provide a quantitative framework for evaluating the n-type performance. Section 3 traces the architectural evolution from dual-through-triple-acceptor to A–A systems, showing how each additional acceptor unit deepens the electronic levels and improves the unipolar behavior. Section 4 connects microstructure to charge transport, highlighting that short-range π-aggregation matters more than bulk crystallinity. Section 5 covers the device engineering, ambient stability—interface treatments, threshold voltage optimization, processing protocols, and operational lifetime assessments. Section 6 discusses emerging opportunities in complementary circuits and bioelectronics alongside persistent challenges, including voltage-stability trade-offs and synthetic limitations. Section 7 concludes that deliberate molecular design combined with interface engineering positions these polymers toward commercial viability.

## 2. Figures of Merit

### 2.1. HOMO/LUMO Energy Level Calculation

**Figure 2:**
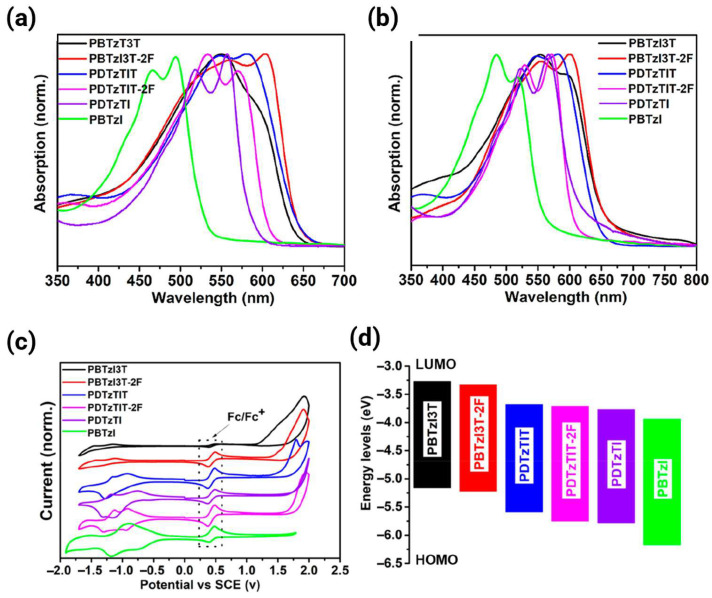
Frontier molecular orbital determination. Normalized UV–vis absorption spectra of polymer semiconductors (**a**) in dilute chloroform solutions (10^−5^ M) showing optical bandgap, and (**b**) as thin films demonstrating solid-state aggregation. (**c**) Cyclic voltammograms of polymer thin films measured in 0.1 M tetrabutylammonium hexafluorophosphate acetonitrile solutions with Fc/Fc^+^ redox couple as internal standard (50 mV s^−1^ scan rate). (**d**) Extracted FMO energy level diagram. Together, these measurements establish LUMO/HOMO levels governing charge injection and unipolar transport characteristics. Reproduced with permission from Ref. [20]. Copyright 2025, American Chemical Society.

The FMO energy levels of HOMO (*E_HOMO_*) and LUMO (*E_LUMO_*) are fundamental to the performance of organic semiconductors, governing the charge injection barriers and ambient stability. Primary methods such as cyclic voltammetry (CV) and optional UV–vis absorption spectroscopy are used to calculate the FMOs by measuring the redox potentials and optical absorption onsets, respectively [2,19]. As a representative example, Figure 2 shows the complementary UV–vis and electrochemical methods used to extract frontier orbital energies, directly connecting the optical absorption onsets and redox potentials to the FMO levels.

#### 2.1.1. Calculation Based on CV [19,20]

FMO energy levels are typically estimated by referencing the potential to the vacuum level. The *E_LUMO_* is derived from the onset reduction potential as follows:

(1) $$E_{LUMO}=-\left(E_{onset}^{red}+E_{ref}\right)$$

Similarly, the *E_HOMO_* is derived from the onset oxidation potential as

(2) $$E_{HOMO}=-\left(E_{onset}^{ox}+E_{ref}\right)$$

where $E_{onset}^{red}$ and $E_{onset}^{ox}$ are the onset reduction and oxidation potentials measured during CV(V), respectively, and *E_ref_* is the absolute energy level of the reference electrode (eV) used to convert the electrochemical potential (measured vs. the reference) to the vacuum level. For ferrocene/ferrocenium (Fc/Fc^+^) redox couple used as an external or internal standard, the common reference value for the absolute energy level to vacuum is −4.80 eV [15,19]. When a saturated calomel electrode is used as the reference electrode, a potential of −4.40 eV has been used to relate the redox potential to the vacuum level [21].

#### 2.1.2. Calculation via Optical Bandgap [17,20]

When oxidation peaks are indiscernible in CV (a common issue for unipolar n-type polymers where the HOMO is very deep), *E_HOMO_* is often calculated using *E_LUMO_* and the optical bandgap ($E_{g}^{opt}$):

(3) $$E_{HOMO}=E_{LUMO}-E_{g}^{opt}\;eV$$

where $E_{g}^{opt}$ is estimated from the onset wavelength ($\lambda_{onset}$) of the thin-film absorption spectrum:

(4) $$E_{g}^{opt}=\frac{1240}{\lambda_{onset}}\;eV$$

#### 2.1.3. Other Methods

In addition to CV and UV–vis spectroscopy, photoelectron emission spectroscopy in air (PESA) and inverse photoemission spectroscopy (IPES) are solid-state techniques used to directly measure the ionization potential (IP) and electron affinity (EA) [8,22].

### 2.2. Electron Mobility

Carrier mobility (µ) is a critical figure-of-merit defining the charge carrier transfer rate and is typically extracted from the current-voltage (I–V) characteristics of the organic field-effect transistor (OFET) in two regimes: linear and saturation. For n-type polymers, µ represents the electron mobility (µ_e_).

#### 2.2.1. Saturation Regime Mobility

In the saturated region, where the drain-source voltage (*V_DS_*) is large (*V_DS_* ≈ *V_GS_* − *V_T_*), the drain current (*I_DS_*) is given by [23].

(5) $$I_{DS}=\frac{W}{2L}{{µ}_{sat}C_{i}\left(V_{G}-V_{T}\right)}^{2}.$$

The saturated mobility (${µ}_{sat}$) can be calculated by fitting the square root of the drain current vs. the gate voltage ($\sqrt{I_{DS}}$ vs. *V_GS_*) plot: [24].

(6) $${µ}_{sat}=\frac{2L}{{WC}_{i}}\;{\left(\frac{\partial \sqrt{I_{DS}}}{\partial V_{DS}}\right)}^{2}$$

where *W* is the channel width, *L* is the channel length, *C_i_* denotes the capacitance per unit area of the gate dielectric layer, *V_GS_* is the gate–source voltage (or gate voltage *V_G_*), and *V_T_* is the threshold voltage (the minimum *V_G_* required to induce the conducting channel).

#### 2.2.2. Linear Regime Mobility

In the linear region, where the *V_DS_* is small (*V_DS_* ≪ *V_GS_* − *V_T_*), I_DS_ has a linear relationship with *V_DS_*: [25].

(7) $$I_{DS}=\frac{W}{L}{µ}_{lin}C_{i}\left(V_{G}-V_{T}\right)V_{DS}$$

#### 2.2.3. Mobility Calculation Methods

The most widely used method is the field-effect transistor (FET) configuration in which the mobility is calculated from the electrical characteristics measured via the output and transfer curves (*I_DS_* vs. *V_DS_* and *I_DS_* vs. *V_GS_*, respectively). It is recommended that both ${µ}_{sat}$ and ${µ}_{lin}$ values be provided for a clear evaluation.

### 2.3. Contact Resistance

The contact resistance (*R_C_*) at the source/drain metal/organic interfaces is crucial, particularly as the channel length decreases, leading to a voltage drop and potential mobility underestimation. In the linear operating regime, the total resistance (*R_tot_*) of the OFET is the sum of the contact and channel resistances (*R_Ch_*) [26].

(8) $$R_{tot}=\frac{V_{DS}}{I_{DS}}=R_{C}+R_{Ch}$$

where *R_Ch_* is the channel resistance and *R_C_W* denote the width-normalized contact resistance. The channel resistance, normalized by the channel width, is given by

(9) $$R_{Ch}W=\frac{L}{\mu_{0}C_{i}(V_{G}-V_{T})}.$$

The width-normalized total resistance (*R_tot_W*) is expressed as

(10) $$R_{tot}W=R_{C}W+R_{Ch}W,$$

where ${µ}_{0}$ is the intrinsic channel mobility (without contact resistance effects).

#### Methods Used to Calculate Contact Resistance

The most common is the transfer line method (TLM) [26]. This involves measuring *R_tot_W* (or *V_DS_*/*I_DS_*) for devices with varying *L* while keeping *V_GS_* constant. Plotting *R_tot_W* vs. *L* yields a straight line whose y-intercept corresponds to *R_C_W* (where *L* = 0) and whose slope is used to determine the intrinsic mobility ${µ}_{0}$. The TLM is the most accurate in the linear regime, with a small voltage dissipation at the contacts.

## 3. Acceptor Design: From Building Blocks to Multi-Acceptor Architectures

### 3.1. Acceptor Building Block Families

The trajectory of high-performance n-type polymer semiconductors is fundamentally constrained by the scarcity of acceptor building blocks that possess sufficiently strong electron-withdrawing capabilities and molecular geometries conducive to optimal charge transport [2,17]. An ideal acceptor unit must simultaneously satisfy several rigorous criteria, that is, it must exhibit deep HOMO and LUMO energy levels, possess a rigid backbone with high coplanarity, and maintain suitable solubility for solution processing [2].

A few distinct families of electron-deficient building blocks have emerged as foundations for n-type polymer development, primarily characterized by imide or amide functionalization.

#### 3.1.1. Imide-Functionalized Arenes

This group, which includes NDIs and perylene diimides (PDIs), offers strong electron-withdrawing characteristics and large surface areas, thereby facilitating efficient interchain charge transport [11,20,27,28]. The copolymer P(NDI2OD–T2), composed of NDI and bithiophene (T2), is a seminal example, being one of the first well-studied high electron mobility materials whose performance exceeded the amorphous silicon benchmark (approximately 0.5–1.0 cm^2^ V^−1^ s^−1^) [10]. Ladder-type polymers, such as the early poly(benzobisimidazobenzophenanthroline) (BBL), also demonstrated electron mobility above 0.1 cm^2^ V^−1^ s^−1^ due to their inherently rigid and planar backbone structures [27,29].

#### 3.1.2. Diketopyrrolopyrrole Derivatives

The diketopyrrolopyrrole (DPP) units are amide derivatives recognized for their planar structure, high crystallinity, and acceptable electron-withdrawing capabilities [2]. DPP-based polymers have been widely incorporated into D–A polymers. However, to achieve unipolar n-type transport (as opposed to common ambipolarity), the acceptor unit often requires further functionalization or dimerization to deepen the FMOs.

#### 3.1.3. Bithiophene Imide Derivatives

**Figure 3:**
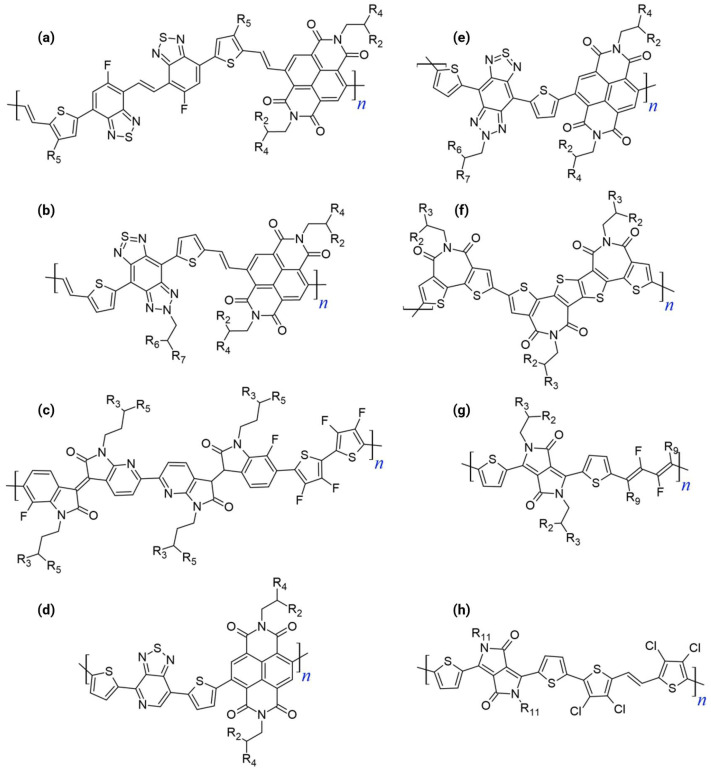
Molecular structures of representative dual-acceptor polymers for n-type PFETs; (**a**) NDI/BBTV/Vinylene—P3 [3], (**b**) NDI/BBTV/Vinylene + SN—P4 [3], (**c**) P2N2F-4BT [21], (**d**) pPTT [31], (**e**) pSNT [31], (**f**) PBTI-BTI2 [32], (**g**) PCNDFDE-DPP [33], and (**h**) DPPTh-4ClTVT [34]; all the above polymers exhibited unipolar n-type character with electron mobilities over 1.0 cm^2^ V^−1^ s^−1^. R_2_ = C_10_H_21_; R_3_ = C_8_H_17_; R_4_ = C_12_H_25_; R_5_ = C_6_H_13_; R_6_ = C_2_H_5_; R_7_ = C_4_H_9_; R_8_ = C_14_H_29_; R_9_ = CN; R_10_ = C_18_H_37_; R_11_ = C_36_H_74_.

**Figure 4:**
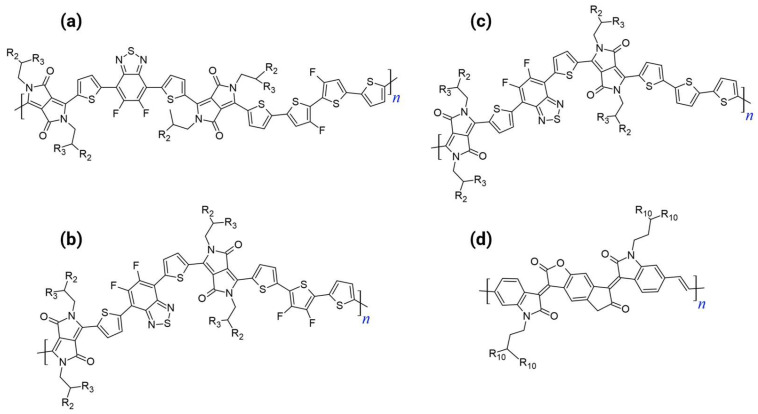
Molecular structures of representative triple-acceptor polymers for n-type PFETs; (**a**) pDFB-2TF [2], (**b**) pDFB-TF [2], (**c**) pDFB-T [2], and (**d**) BDPPV [18]; all the above polymers exhibited unipolar n-type character with electron mobilities over 1.0 cm^2^ V^−1^ s^−1^. R_2_ = C_10_H_21_; R_3_ = C_8_H_17_; R_4_ = C_12_H_25_; R_5_ = C_6_H_13_; R_6_ = C_2_H_5_; R_7_ = C_4_H_9_; R_8_ = C_14_H_29_; R_9_ = CN; R_10_ = C_18_H_37_; R_11_ = C_36_H_74_.

**Figure 5:**
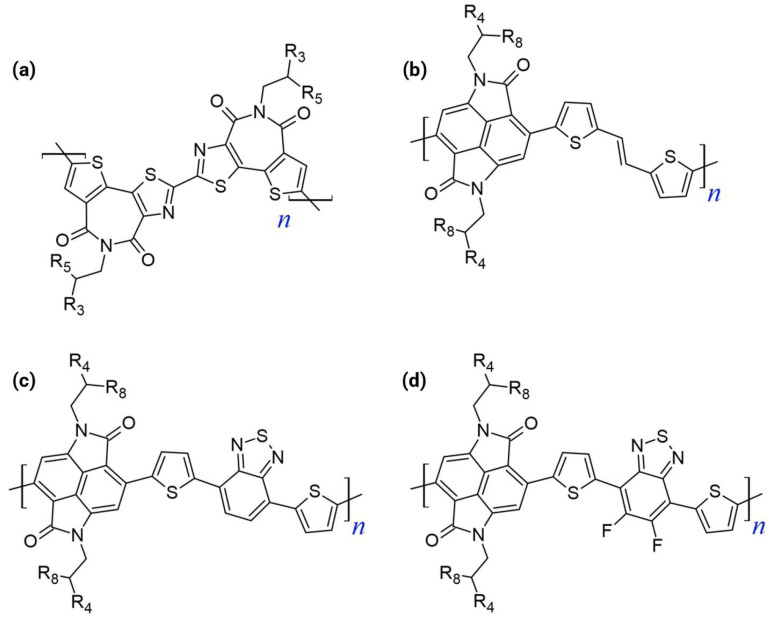
Molecular structures of representative all-acceptor polymers for n-type PFETs; (**a**) PDTzTI [4], (**b**) (NBA-TVT)_4_—P2 [10], (**c**) (NBA-DTBT)_4_—P3 [10], and (**d**) (NBA-DTBTff)_4_—P4 [10]; all the above polymers exhibited unipolar n-type character with electron mobilities over 1.0 cm^2^ V^−1^ s^−1^. R_2_ = C_10_H_21_; R_3_ = C_8_H_17_; R_4_ = C_12_H_25_; R_5_ = C_6_H_13_; R_6_ = C_2_H_5_; R_7_ = C_4_H_9_; R_8_ = C_14_H_29_; R_9_ = CN; R_10_ = C_18_H_37_; R_11_ = C_36_H_74_.

Bithiophene imide (BTI, N-alkyl-2,2′-bithiophene-3,3′-dicarboximide) is a crucial framework for multi-acceptor design [20,30]. BTI offers a low-steric-hindrance profile and intrinsic core planarity. Despite these advantages, BTI-based polymers initially suffered from a “high-lying LUMO issue” due to the electron-rich thiophene segment, which limits their intrinsic n-type stability and performance. This specific limitation necessitates advanced acceptor-based strategies, driving research toward multi-acceptor architectures to further deepen the LUMO level [17]. The structural diversity of these polymers is illustrated in Figure 3, Figure 4 and Figure 5, which shows repeat unit structures spanning dual-acceptor, triple-acceptor, and A–A designs. The progression from dual-acceptor to triple-acceptor to A–A architectures is presented in Table 1, which collates the acceptor building blocks, FMO levels determined via CV, synthesis methodologies, and molecular weight distributions across representative systems.

### 3.2. Dual-Acceptor Strategy: Foundation of All-Acceptor Approach

#### 3.2.1. Design Rationale

The dual-acceptor strategy serves as a critical bridge between conventional D–A systems and the optimal A–A paradigm. This methodology deliberately increases the relative content of the electron-deficient moieties in the polymer backbone (e.g., forming an A_1_–A_2_ or D–A_1_–A_2_ repeating unit). Figure 3 shows the chemical structures of the dual-acceptor n-type polymers.

The design rationale centers on electronically and physically manipulating the FMOs:

- FMO Deepening: LUMO energy level was significantly lowered (or deepened) by enhancing the electron-withdrawing capacity by incorporating two acceptor units [17,35]. A deep LUMO (ideally near or below −4.0 eV) is essential as it facilitates efficient electron injection from the electrode and stabilizes the resulting electron transport, thus mitigating instability issues caused by ambient oxygen and water [33,35,36]. Concurrently, deepening the HOMO level increases the ionization potential, effectively suppressing hole injection, and guaranteeing the desired unipolar n-type characteristics with minimized minority carrier transport.
- Minimizing ICT: The dual-acceptor approach inherently suppresses the ICT characteristic observed between neighboring donor and acceptor units in D–A polymers. The reduced ICT promotes higher electron affinity and stabilizes the resulting polymers [29,32,36].
- Enhancing Microstructure: Including multiple acceptors, especially those containing heteroatoms, can enhance intermolecular interactions (such as π-π stacking or heteroatom contacts), contributing to a more highly ordered molecular packing and improved charge hopping pathways [37].

#### 3.2.2. Synthesis Methods for Dual-Acceptor Systems

The syntheses of dual-acceptor and A–A polymers are intrinsically more challenging than those of their D–A counterparts. Electron-deficient monomers often exhibit poor stability and low reactivity during polymerization, typically resulting in a low molecular weight (M_n_).

- Stille Polycondensation: Palladium-catalyzed Stille coupling is the standard protocol. However, reactivity issues often arise when two highly electron-deficient monomers are coupled. For instance, the polymerization of dibrominated BTzI monomer (N-alkyl-5,5′-bithiazole-4,4′-dicarboximide) required the use of copper iodide (CuI) as a co-catalyst to achieve the desired A–A homopolymer (PBTzI) with a moderate molecular weight (5.8 kDa) [20].
- Specialized Monomers: Innovations in monomer synthesis are crucial. The use of a specialized monomer, such as distannylated bithiophene imide (BTI-Tin), has successfully enabled the formation of high-performance A–A backbone semiconductors, such as PBTI and the A–A copolymer P(BTI-BTI_2_) [32].

### 3.3. Triple-Acceptor Architecture: Breakthrough to Ultra-High Performance

#### 3.3.1. Three Acceptors: Strategic Advantages

The progression to a triple-acceptor architecture represents an aggressive extension of the multi-acceptor design philosophy aimed specifically at overcoming the residual shortcomings in the FMO levels or structural order that persist even in dual-acceptor systems. Figure 4 shows the chemical structures of the triple-acceptor n-type polymers. The introduction of three acceptor units is highly strategic for achieving unipolar n-type transport.

- Maximal Electron Affinity: The introduction of three strongly electron-deficient units maximizes the electron-withdrawing effect across the polymer repeat units. This maximized deficiency significantly deepened the LUMO and HOMO levels, leading to superior electron injection properties and complete elimination of hole injection, thereby achieving purer unipolar n-type characteristics [37].
- Hybrid Modulation for Unipolarity: Truly successful high-performance n-type polymers often rely on a hybrid strategy that combines multiple acceptors with strategic functionalization. The triple-acceptor architecture provides ample opportunities to incorporate multiple structure-tailoring groups via fluorination or cyanation into different parts of the overall acceptor unit. Fluorination is particularly effective, as the fluorine atoms can induce rigid backbones and high coplanarity through F∙∙∙H or F∙∙∙S non-covalent interactions [2,38].
- Optimized Charge Transport: It is posited that the three sequential acceptors may enhance the intramolecular properties by inducing greater charge coupling, which could lead to a smaller effective mass (m*) of the charge carriers, ultimately promoting more efficient ICT.

#### 3.3.2. Case Study (Unipolar n-Type Focus)

A dedicated example of this strategic approach is a fluorinated triple-acceptor architecture developed for high-performance unipolar n-type organic transistors. This architecture involves the substitution of fluorine within a complex multi-acceptor structure (leading to the polymer pDFB-TF) [2]. This hybrid acceptor modulation strategy was considered a feasible approach to achieve improved unipolar n-type charge-transporting properties, specifically addressing the issue in which previous DPP dimerization/trimerization strategies failed to maintain a unipolar character because of the shallow HOMO levels caused by other linking units.

#### 3.3.3. Synthesis Methods for Triple-Acceptor Systems

Although detailed generalized synthesis methods for triple-acceptor systems are scarce, the complexity of this architecture necessitates robust and controlled cross-coupling methods typically involving advanced Pd-catalyzed polycondensation techniques [2]. The successful synthesis of these intricate structures requires meticulously designed multifunctional monomers, often employing halogenated and organostannane (Sn) or organoboronic (B) derivatives for Stille and Suzuki couplings, respectively. Given the enhanced electron deficiency, the challenges of low reactivity and instability (as noted for dual-acceptor systems) are likely exacerbated, placing a high demand on the quality and stability of the constituent monomers.

### 3.4. All-Acceptor Architecture

The A–A architecture simplifies the molecular design principle by eliminating all electron-donating units and constructing a conjugated polymer backbone purely from electron-deficient moieties.

#### 3.4.1. Strategic Advantages

The A–A strategy is fundamentally superior to the D–A approach for developing unipolar n-type polymers, leading to materials with ideal transistor characteristics. Figure 5 shows the chemical structures of the representative all-acceptor n-type polymers.

- Suppression of Ambipolarity: The A–A structure successfully suppresses the ICT, leading to extremely deep FMOs (both LUMO and HOMO) [29,35,36]. Deep LUMOs facilitate electron transport, whereas deep HOMOs efficiently block hole injection.
- Ideal Device Performance: The dual FMO control results in superior device figures of merit, including ultralow off-currents (I_off_ in the range of 10^−10^–10^−11^ A) and substantial current on/off ratios (I_on_/I_off_ typically 10^7^–10^8^) [4,29]. In contrast, high-mobility D–A copolymers often suffer from ambipolar transport with a high I_off_ (>10^8^ A) and small I_on_/I_off_ (<10^5^) [4]. The ability of the A–A approach to achieve unipolar electron transport with nearly ideal characteristics underscores its significance.
- Enhanced Electron Affinity and Stability: The highly electron-deficient nature imparted by the A–A backbone increases electron affinity, which is crucial for ambient device operational stability, especially compared to D–A systems, where the donor units destabilize the LUMO.

#### 3.4.2. Synthesis Methods for All-Acceptor Systems

The inherent synthetic difficulties associated with handling highly electron-deficient monomers require specialized polymerization protocols for A–A materials.

- Optimized Stille Polycondensation: This method has proven to be effective when highly purified monomers are used. In the synthesis of ladder-type homopolymers of PBTIn, Pd-catalyzed Stille coupling yielded superior material quality and higher molecular weights than Ni-mediated Yamamoto coupling, directly correlating with improved device performance [29].
- Direct Arylation Polycondensation: Direct arylation and polycondensation (DArP) offers an atom-economical and environmentally friendly synthesis [38]. A modified DArP method, typically employing Pd/Cu co-catalysts, successfully produced high-molecular-weight A–A polymers, even when the monomers lacked the conventional orienting or activating groups required for C–H bond activation. This protocol successfully produced high-quality A–A polymers that were previously inaccessible via the conventional Suzuki or Stille methods [35].

### 3.5. Multi-Acceptor Performance Hierarchy and Design Guidelines

#### 3.5.1. Design Guidelines

The successful implementation of multi-acceptor strategies is dictated by two overarching goals: tuning the electronic structure for unipolarity and optimizing the physical structure for efficient transport.

- Electronic Structure Control (Deep FMOs): The central tenet of unipolar n-type polymers is deep LUMO to facilitate electron injection/transport and concurrently deep HOMO to suppress hole injection [3,33]. This is systematically achieved by increasing the overall loading of electron-withdrawing units, as exemplified by the shift from D–A to A–A architectures and the incorporation of strong electron-withdrawing functionalities (e.g., cyano or fluorine substitution) onto the acceptor backbone [2,17].
- Solubility and Molecular Weight: To enable solution processing for thin-film fabrication, sufficient alkyl side chains must be incorporated to ensure solubility, even as the extended backbone decreases the intrinsic solubility [37]. Furthermore, a high molecular weight is crucial because the charge transport properties can be sensitive to the degree of polymerization. Therefore, it is necessary to develop tailored synthesis methods to achieve a high M_n_ for inherently low-reactivity A–A monomers [29].

#### 3.5.2. When Does Complexity Pay Off?

Complexity in acceptor design, encompassing multi-acceptor structures, fused rings, and peripheral functionalization, pays off primarily when energetic disorder is successfully managed and favorable molecular packing is dictated, which is a critical factor that often exceeds the importance of raw electronic performance metrics.

- Payoff of Hybrid Modulation: The productive complexity was observed in the hybrid approach (multi-acceptor + strong EWGs). For example, the cyanofunctionalization of BTI (creating CNI derivatives) overcomes the performance ceiling of the unfunctionalized BTI acceptor, yielding significant improvements in the n-type stability and FMO depth [17].
- Diminishing Returns of Ladder Extension: Conversely, the complexity of extending the fused ladder-type structure does not always yield proportional performance gains. In the A–A homopolymer series PBTIn, increasing the monomer size from PBTI1 (3 rings, µ_e_ = 3.71 cm^2^ V^−1^ s^−1^) to PBTI5 (15 rings, µ_e_ = 0.014 cm^2^ V^−1^ s^−1^) resulted in a monotonic two-order-of-magnitude decrease in electron mobility [29]. This decrease was attributed to the extension of the ladder-type blocks, which negatively affected the freedom of motion of the building blocks and polymer chains during film formation, leading to increased disordered phases, reduced crystallinity, and diminished charge transport. This result serves as a key guideline: maximizing the conjugation and rigidity does not inherently lead to better mobility if it compromises the ability of the material to self-assemble into a highly ordered film.

### 3.6. Halogenation Strategy

The halogenation strategy by the addition of fluorine or chlorine to the backbone of conjugated polymers causes multiple effects. This strategy is primarily used for modulating the FMOs and enhancing the ambient stability in n-type polymers. However, there are additional negative effects such as the introduction of conformal constraints, which arise from the non-covalent interactions. These effects can be balanced by choice of the halogen atom (fluorine or chlorine) and/or the substitution position in the backbone.

Fluorination has been a widely studied strategy due to its exceptional electronegativity (3.98 on the Pauling scale) and smaller van der Waals radius (1.47 Å). Multifluorination strategy has also been studied, in which both the donor and acceptor units are fluorinated. Gao et al. synthesized a series of polymers (P0F through P6F) containing 0 to 6 fluorine atoms per repeating unit, revealing systematic depression of frontier orbitals. As reported, the LUMO levels decreased from −3.63 eV (P0F) to −3.83 eV (P6F) while HOMOs dropped from −5.48 to −5.82 eV [39]. This reduction in the HOMO level altered the charge injection characteristics of the synthesized polymers in such a way that, P0F exhibited unipolar p-type behavior, P2F and P4F displayed ambipolar characteristics, and P6F achieved balanced ambipolar transport with μₕ = 3.94 cm^2^ V^−1^ s^−1^ and μₑ = 3.50 cm^2^ V^−1^ s^−1^. On top of these, moving the alkyl branching point away from the backbone in P6F-C3 further depressed the HOMO to −5.95 eV, which completely suppressed the hole injection to yield unipolar n-type behavior with μₑ reaching 4.97 cm^2^ V^−1^ s^−1^. It is therefore demonstrated that the halogenation strategy, when synergized with side-chain engineering, can not only increase carrier mobility but also alter polarity. There are also significant conformational effects due to fluorination. A reduction in the torsion angle from 19.80° (P0F) to 0.24° (P6F) was reported as a consequence of the F···H (2.16 Å) and F···S (2.70 Å) noncovalent interactions. This planarity translated to tighter π-stacking (3.48 Å for P2F versus 3.73 Å for P0F) and enhanced intermolecular coupling. Critically, multifluorination delivered exceptional ambient stability—P6F-based devices retained μₑ = 0.05 cm^2^ V^−1^ s^−1^ after 60 days in ambient conditions (RH 20–40%), whereas P2F and P4F mobilities degraded one order of magnitude within 4 days [39]. This stability stems directly from the lowered LUMO resisting electron capture by atmospheric oxygen and water during device operation.

Chlorination offers an intriguing alternative despite chlorine’s larger atomic radius (1.75 Å). Wei et al. systematically compared fluorinated and chlorinated naphthalene diimide (NDI) copolymers (N2200, F-N2200, Cl-N2200), uncovering position-dependent effects that diverge from fluorination trends [40]. Fluorination reduced energetic disorder in N2200, lowering activation energy and modestly improving transport despite slightly reduced mobility. Chlorination, conversely, increased energetic disorder due to more twisted backbone conformations, i.e., Cl-N2200 exhibited the highest disorder yet both F-N2200 and Cl-N2200 demonstrated lower mobility values compared to the pristine N2200. However, Cl-N2200 showed the strongest response to molecular doping, with mobility increasing from 5.57 × 10^−3^ to 1.39 × 10^−2^ cm^2^ V^−1^ s^−1^ upon 1 wt% TBABr doping [40]. This paradoxical behavior reflects chlorine’s dual character: backbone chlorination often blueshifts absorption and decreases planarity through steric hindrance, yet side-chain or terminal chlorination can enhance crystallinity and energy level alignment without disrupting backbone coplanarity [41].

The position-specificity of chlorination creates architectural opportunities unavailable through fluorination. Terminal chlorination in benzodithiophene (BDT) derivatives and dicyanoindene (DCI) end groups effectively lowers LUMO levels (often by 0.1–0.2 eV) while maintaining or even improving crystallinity when steric conflicts are avoided [41]. This distinction enables chlorinated acceptor units like 2Cl-DCI to combine deep frontier orbitals with tight molecular packing—a balance difficult to achieve through backbone halogenation alone. The synthetic accessibility of chlorination (simpler reactions, higher yields, lower cost than fluorination) further positions it as a practical complement to fluorination strategies, particularly for large-scale applications where economic constraints matter. Both approaches demonstrate that strategic halogenation transcends simple electron withdrawal, offering multidimensional control over electronics, conformation, and ambient resilience essential for unipolar n-type operation.

### 3.7. Cyano Functionalization Strategy

Cyano group incorporation stands as one of the most powerful electron-withdrawing strategies for n-type polymer development, offering LUMO depression exceeding 0.5 eV in favorable cases, yet success hinges critically on positioning these sterically demanding substituents to avoid catastrophic backbone twisting. The triple-bonded nitrile moiety (C≡N) exerts exceptionally strong inductive electron withdrawal (Hammett constant σₘ = +0.56), fundamentally altering frontier orbital distributions and charge injection characteristics—but the approximately 1.55 Å C–C≡N bond length coupled with the linear geometry creates steric conflicts when positioned improperly, destroying the planarity essential for π-orbital overlap [42,43,44].

**Figure 6:**
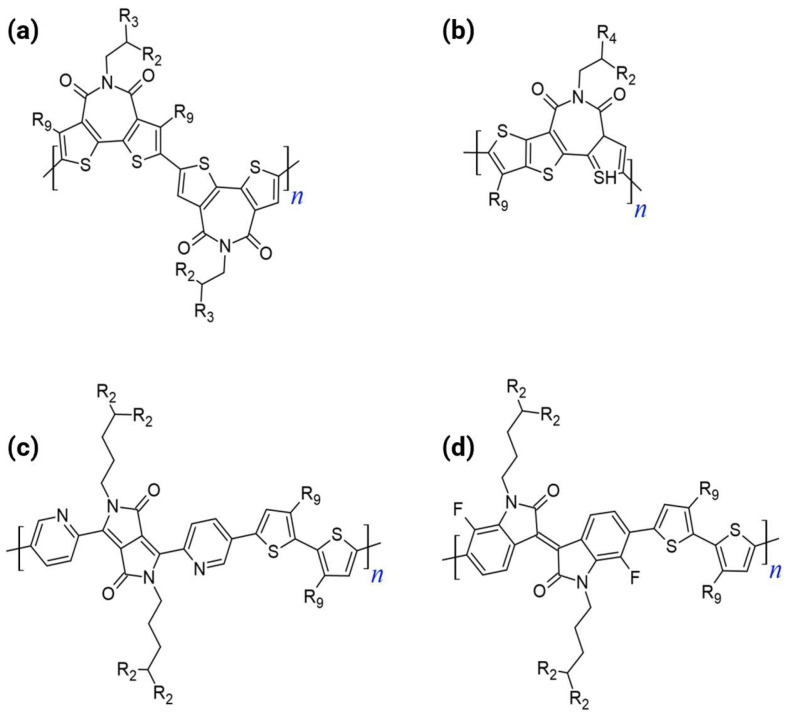
Molecular structures of representative cyano-functionalized polymers for n-type PFETs; (**a**) PCNI-BTI [17], (**b**) PCNTI [17], (**c**) DPPPy-BT2CN [45], and (**d**) 2FIIDCN [45]; R_2_ = C_10_H_21_; R_3_ = C_8_H_17_; R_4_ = C_12_H_25_; R_5_ = C_6_H_13_; R_6_ = C_2_H_5_; R_7_ = C_4_H_9_; R_8_ = C_14_H_29_; R_9_ = CN; R_10_ = C_18_H_37_; R_11_ = C_36_H_74_.

The positioning paradox emerged starkly in stilbene-based systems. Direct cyano substitution on aromatic rings invariably increases dihedral angles; DFT calculations revealed torsion angles escalating from 0.15° (unsubstituted) to 27.03° (mono-cyano) and 38.87° (di-cyano) between benzene and vinylene units in stilbene derivatives [43]. This twisting disrupts effective conjugation length and fragments the π-system, offsetting any electronic gains from LUMO lowering. Chen and colleagues synthesized three isoindigo-stilbene copolymers (P0, P1, P2) with increasing cyano content, observing systematic transitions: P0 exhibited p-type behavior (μ_h_ = 0.40 cm^2^ V^−1^ s^−1^) with LUMO at −3.96 eV, while mono-cyano P1 achieved n-type transport (μ_e_ = 0.62 cm^2^ V^−1^ s^−1^, LUMO −4.17 eV) and di-cyano P2 further improved performance (μ_e_ = 0.83 cm^2^ V^−1^ s^−1^, LUMO −4.25 eV) [43]. Critically, the HOMO levels dropped precipitously from −5.31 eV (P0) to −5.85 eV (P2)—a 0.54 eV depression that eliminated hole injection from gold electrodes, converting the material from p-type to unipolar n-type. This demonstrates cyano functionalization’s unique capacity for simultaneous polarity switching and mobility enhancement when backbone planarity is preserved. Figure 6 shows the chemical structures of the representative cyano-functionalized n-type polymers.

The thienylene-vinylene-thienylene (TVT) unit emerged as the architectural solution to the planarity constraint. Thiophene’s compact five-membered ring generates substantially less steric hindrance than benzene during vinylene cyano substitution; calculations confirmed that mono- and di-cyano TVT derivatives maintain near-perfect planarity (dihedral angles < 1°) between thiophene and vinylene segments [44]. This planarity preservation proves mechanistically crucial—it creates a donor (thiophene)-acceptor (cyanated vinyl)-donor structure with minimized push-pull distances, strengthening intramolecular charge transfer while maintaining extended conjugation. Zhang and coworkers systematically compared mono-cyano (M1) and di-cyano (M2) TVT monomers copolymerized with diketopyrrolopyrrole, revealing that M2’s double substitution generated more balanced donor-acceptor distributions along the backbone [44]. Electrostatic potential mapping demonstrated that M2 formed a distinct D-A_1_-D-A_2_ motif where thiophene donors (positive potential) were separated by cyano-vinylene acceptors (negative potential −40.16 to −40.39 kcal/mol), creating sharper electronic gradients than M1’s quasi-continuous donor regions. Polymer P2 (based on M2) achieved μ_e_ = 1.03 cm^2^ V^−1^ s^−1^—nearly double P1’s 0.52 cm^2^ V^−1^ s^−1^—with LUMO deepening from −3.55 to −3.80 eV [44]. GIWAXS analysis confirmed that enhanced cyano substitution promoted tighter molecular packing and improved long-range order after thermal annealing, validating that strategic positioning converts steric liabilities into packing assets.

The combination of cyano functionalization with other acceptor moieties unlocked performance breakthroughs. Feng and colleagues incorporated cyano-functionalized TVT (TVTCN) alongside fused bithiophene imide dimer (f-BTI_2_) to synthesize f-BTI_2_g-TVTCN, a polymer exhibiting record n-type organic electrochemical transistor performance (μ_e_,OECT = 0.24 cm^2^ V^−1^ s^−1^, volumetric capacitance C* = 170 F cm^−3^) [42]. The cyanation lowered both LUMO (−3.81 eV) and HOMO (−5.57 eV) by approximately 0.3 eV relative to the non-cyanated analog f-BTI_2_g-TVT, while DFT calculations confirmed backbone planarity preservation (torsion angle < 0.1°).

Crucially, the cyano groups enhanced polaron delocalization—the LUMO distribution spread more uniformly across the cyano-vinylene segments, reducing reorganization energy and promoting efficient charge hopping. The non-cyanated control achieved only μ_e_,OECT = 0.014 cm^2^ V^−1^ s^−1^, demonstrating an order-of-magnitude improvement solely attributable to strategic cyano placement [42]. This finding extends beyond transistors; cyano-functionalized polymers with sufficiently deep LUMOs (−3.7 to −4.0 eV) resist ambient oxidation, maintaining performance for months without encapsulation—a stability profile unattainable through donor-acceptor architectures alone. The strategic lesson crystallizes: cyano functionalization demands positional precision, with vinylene linkages in compact heterocycles like TVT offering the optimal compromise between electron withdrawal strength and structural integrity maintenance.

### 3.8. Doping Strategy for High-Performance n-Type Polymers

Molecular doping represents a transformative strategy for n-type polymer performance enhancement, transcending the intrinsic mobility limitations imposed by pristine semiconductors through controlled carrier injection—yet success demands precise energetic alignment between dopant and host alongside microstructural compatibility that preserves charge transport pathways. Unlike conventional electronic doping, where impurity atoms substitute into crystalline lattices, organic molecular doping introduces discrete dopant molecules that must diffuse into the amorphous or semi-crystalline polymer matrix, transfer electrons to the LUMO manifold, and avoid disrupting the delicate π-stacking architecture essential for interchain hopping [46,47]. The process fundamentally alters device physics: doped films exhibit dramatically increased conductivity (σ = nqμ, where n is carrier density and μ is mobility) by raising n from ~10^15^ cm^−3^ in pristine films to 10^18^–10^20^ cm^−3^ upon effective doping, while simultaneously introducing Coulomb interactions between mobile carriers and ionized dopants that can paradoxically reduce mobility through scattering [46,47].

The hydride-transfer dopant N-DMBI (4-(1,3-dimethyl-2,3-dihydro-1H-benzoimidazol-2-yl)-N,N-dimethylaniline) emerged as the most successful n-type molecular dopant, operating through a distinct mechanism from simple electron transfer. Mechanistic studies by Naab and colleagues revealed that N-DMBI functions via bimolecular hydride transfer to the acceptor polymer in the rate-determining step, followed by electron transfer from the resulting DMBI radical to generate polymer radical anions and stable DMBI cations [48]. This mechanism proves advantageous because doping efficacy depends not solely on HOMO (N-DMBI) and LUMO (polymer) alignment, but rather on the thermodynamics of hydride abstraction—a pathway that can proceed even when direct electron transfer is energetically unfavorable. Cyano-functionalized imide-based polymers demonstrated exceptional compatibility with N-DMBI doping; PCNDTI with its deep LUMO (−4.11 eV) achieved conductivity of 0.56 S cm^−1^, representing two orders of magnitude improvement over the parent PBTI (σ = 2.0 × 10^−3^ S cm^−1^) [17]. The doping mechanism was confirmed through UV–vis-NIR spectroscopy showing suppressed neutral π-π* transitions concurrent with strong sub-bandgap polaronic absorption bands extending to 2500 nm, characteristic of successful electron transfer [17].

Sequential doping—where the polymer film is deposited and optimized independently before dopant application from an orthogonal solvent—emerged superior to blend doping for preserving microstructural order. This two-step approach allows polymer chains to adopt optimal packing configurations during initial film formation, after which dopant molecules permeate the amorphous domains without disrupting crystalline π-stacking regions [17]. Sequential processing proved particularly critical for cyano-functionalized n-type polymers. For the low-crystallinity polymer PCNI-BTI, sequential doping—where the polymer film is cast first followed by N-DMBI-H dopant application from an orthogonal solvent—yielded significantly higher electrical conductivity (σ = 22.4 S cm^−1^) compared to blend doping (σ = 7.2 S cm^−1^). This advantage stems from two factors: first, low-crystallinity polymers accommodate dopant molecules more readily in their amorphous regions without disrupting existing order; second, sequential processing allows independent optimization of polymer film deposition conditions for maximum mobility before introducing dopants, thereby preserving beneficial morphological features that blend doping would disrupt [17].

An alternative doping pathway emerged through proton abstraction using alkali metal carbonates, particularly Cs_2_CO_3_. Hochgesang and coworkers demonstrated that CO_3_^2−^ anions deprotonate labile thiophene protons on the acceptor–acceptor polymer poly(DPP-TPD) during thermal annealing, shifting the Fermi level toward the polymer LUMO without requiring hydride transfer [49]. This mechanism proved remarkably effective—Cs_2_CO_3_-doped poly(DPP-TPD) achieved conductivity performance rivaling N-DMBI while offering superior scalability through lower cost and simpler synthesis. The doping efficacy scaled with carbonate basicity and alkali metal ion radius, with larger cations (Cs^+^) providing better performance than smaller ones (Li^+^, Na^+^) [49]. Thermoelectric measurements revealed power factors reaching (5.6 ± 0.39) × 10^−6^ W m^−1^ K^−2^ for carbonate-doped systems—performance competitive with conventional organic dopants while utilizing earth-abundant, inexpensive materials. Critically, X-ray diffraction studies confirmed that neither N-DMBI nor Cs_2_CO_3_ doping altered short-range polymer microstructure, suggesting dopants reside primarily in fully amorphous domains where they passivate trap states without disrupting ordered π-aggregates [49].

The trap-passivation effect of doping carries particular significance for polymers with high energetic disorder. Wei’s systematic comparison of fluorinated and chlorinated N2200 revealed that Cl-N2200, despite possessing the highest intrinsic disorder, exhibited the strongest doping response—mobility increased from 5.57 × 10^−3^ to 1.39 × 10^−2^ cm^2^ V^−1^ s^−1^ upon 1 wt% N-DMBI doping [40]. In contrast, F-N2200 with inherently low disorder showed minimal mobility enhancement from doping, indicating that dopant-induced carriers primarily fill existing trap states rather than creating new transport channels [40]. This finding establishes a design guideline: polymers intentionally engineered with deep LUMOs and moderate disorder may paradoxically outperform perfectly ordered systems after doping, as the combination of facile dopant infiltration and effective trap passivation yields conductivities approaching metallic regimes while maintaining reasonable Seebeck coefficients for thermoelectric applications. The convergence of molecular design, dopant chemistry, and processing methodology positions doped n-type polymers to achieve conductivities exceeding 100 S cm^−1^—performance approaching conjugated radical polymers while retaining solution processability and ambient stability essential for practical implementation.

## 4. Microstructure, Transport, and Device Engineering

### 4.1. Charge Transport Mechanisms

Electron transport in conjugated polymers occurs via two distinct pathways: intra-chain delocalization along the conjugated backbone and interchain hopping between adjacent molecules through π-orbital overlap. Neither pathway suffices for high mobility, and efficient devices require both. The intramolecular pathway, which is essentially band-like transport within a single molecule, determines how far an electron can travel before transferring to a neighboring chain. This is often quantified using effective mass m*, where smaller values indicate stronger delocalization and more facile intrachain movement. However, the polymer chains are finite and rarely aligned in an end-to-end manner. Consequently, long-range transport inevitably demands interchain hopping, where electrons tunnel quantum mechanically through the van der Waals gap separating neighboring π-systems [23,50,51].

The rate of this interchain transfer follows Marcus-Hush theory, in which the hopping rate depends exponentially on both the reorganization energy *λ* (energy cost of structural relaxation upon charge transfer) and the electronic coupling transfer integral *V* (strength of wave function overlap between adjacent chains) [2].

(11) $$K=\frac{2\pi }{h}V^{2}{\left[\frac{1}{\left(4\pi k_{B}T\lambda \right)}\right]}^{0.5}\exp\left[\frac{-{\left({∆G}^{0}+\lambda \right)}^{2}}{4\lambda k_{B}T}\right]$$

where *T* is temperature, *k_B_* is Boltzmann constant, ℏ is reduced Planck constant, and Δ*G*^0^ is the reaction free energy.

**Figure 7:**
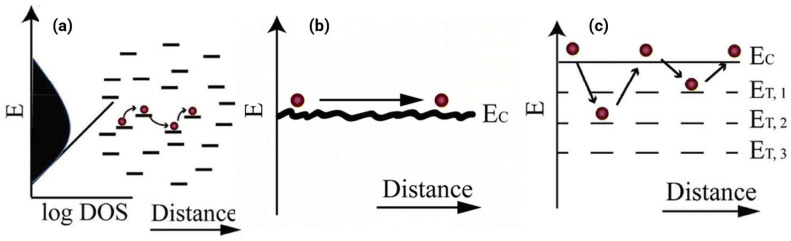
Charge transport mechanisms in polymer field-effect transistors. (**a**) Hopping transport showing density of states (DOS) disorder with carriers jumping between localized states; (**b**) band-like conduction with minimal energetic disorder and extended states; (**c**) multiple trapping and release (MTR) mechanism where charges move through extended states until trapped by defects at different trap energy levels (E_T,1_, E_T,2_, E_T,3_). Red sphere represents the electron. Reproduced with permission from Ref. [52]. Copyright 2025 Royal Society of Chemistry.

For pDFB-TF, a polymer with full conformational locks enforcing backbone rigidity, the reorganization energy is minimum because the molecular geometry barely changes upon oxidation or reduction. The relationship among the carrier mobility, reorganization energy, and transfer integral can be described using the semi-classical charge transport equation, where the mobility is inversely proportional to the reorganization energy and proportional to the square of the transfer integral. Simultaneously, close π-π stacking such as 3.62 Å—maximizes electronic coupling by enhancing wavefunction overlap. Together, these factors enable pDFB-TF to achieve µ_e_ exceeding 5 cm^2^ V^−1^ s^−1^, one of the highest reported for unipolar n-type polymers [2]. Figure 7 compares three transport regimes—hopping between localized states, band-like conduction, and multiple trapping-release cycles—with the Marcus-Hush theory, quantifying the competition between electronic coupling and reorganization barriers.

Temperature-dependent measurements provide critical insights into transport mechanisms. Most n-type polymers exhibit thermally activated mobility following μ(T) = μ exp(−Ea/kT), with activation energies spanning 20–200 meV [53,54]. This positive temperature coefficient indicates hopping transport, where thermal energy helps carriers overcome the energetic barriers between localized states. Lower Ea values (approximately 20–50 meV) indicate reduced energetic disorder and more uniform site energies, edging toward band-like behavior.

Multiple trapping and release (MTR) provide an alternative framework for understanding transport. Here, the charges move freely through the extended states until they are trapped by structural or chemical defects and require thermal activation for release [54]. The density and depth of trap states profoundly influence mobility: shallow traps (Et ~0.1–0.2 eV) are easily thermally depopulated at room temperature, whereas deep traps (Et > 0.3 eV) can immobilize carriers for extended periods. The A–A design paradigm inherently targets deep LUMO levels (often below −4.0 eV) to suppress electron traps associated with ambient oxygen and water, while simultaneously maintaining deep HOMOs to prevent hole injection—the energetic foundation of unipolar n-type operation.

Molecular design strategies directly target these mechanisms. Non-covalent conformational locks—F···S, O···H, and S···O interactions—enforce planarity and minimize reorganization energy [2,4]. Cyano-functionalized acceptors simultaneously deepen the frontier orbitals and maintain backbone coplanarity by reducing the steric hindrance [45]. The polymer pDFB-TF exemplifies this design philosophy. Its fully locked conformation creates a shape-persistent backbone with minimal torsional disorder, enabling both efficient intrachain delocalization and strong interchain coupling [5].

### 4.2. Molecular Packing and π-Stacking

Having established that interchain hopping critically determines device-level mobility, we now examine how molecules are arranged in the solid state to enable or frustrate this process. Grazing-incidence wide-angle X-ray scattering (GIWAXS) serves as the primary characterization tool, revealing both the π-stacking distance (d_π-π_) and the CCL—two parameters that directly modulate electronic coupling strength and transport pathway connectivity.

**Figure 8:**
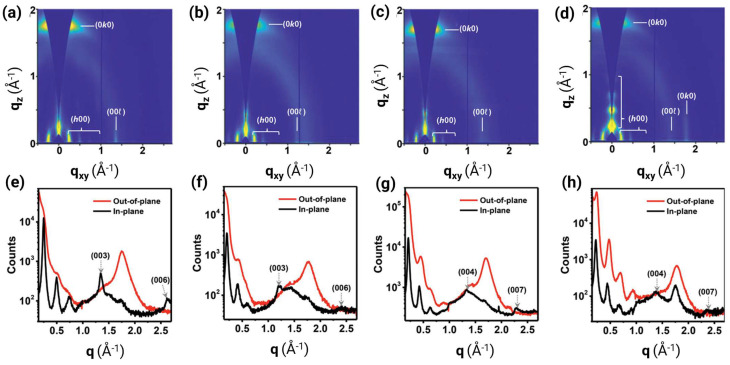
Molecular packing characterized by GIWAXS and its correlation to electron transport. 2D GIWAXS patterns and corresponding 1D scattering profiles (in-plane and out-of-plane) for multi-acceptor polymer films: (**a**,**e**) P1 = P(NBA2DH-T2), (**b**,**f**) P2 = P(NBA2DH-TVT), (**c**,**g**) P3 = P(NBA2DH-DTBT), and (**d**,**h**) P4 = P(NBA2DH-DTBTff). The comonomer series systematically increases backbone rigidity and planarity from bithiophene (T2) through thienylenevinylene-thiophene (TVT) to difluorinated dithienylbenzothiadiazole (DTBTff). Structural parameters extracted from Scherrer analysis: P1 exhibits d_π-π_ = 3.58 Å with crystalline coherence length (CCL) = 40.8 Å (~11 stacked chains), μ_e_ = 0.32 cm^2^ V^−1^ s^−1^; P2 shows d_π-π_ = 3.55 Å, CCL = 38.1 Å (~11 chains), μ_e_ = 1.33 cm^2^ V^−1^ s^−1^; P3 displays d_π-π_ = 3.63 Å, CCL = 37.2 Å (~10 chains), μ_e_ = 2.67 cm^2^ V^−1^ s^−1^; P4 demonstrates d_π-π_ = 3.53 Å with bimodal orientation—CCL = 40.8 Å (12 chains face-on) plus 61.5 Å (17 chains edge-on), μ_e_ = 3.73 cm^2^ V^−1^ s^−1^. Reproduced with permission from Ref. [10]. Copyright 1999–2025 John Wiley & Sons, Inc.

For efficient interchain hopping, a sufficiently close π-π contact is non-negotiable. The exponential dependence of the electronic coupling on the intermolecular separation indicates that sub-angstrom differences in d_π-π_ translate into order-of-magnitude mobility variations. High-performance n-type polymers consistently demonstrate remarkably tight packing with d_π-π_ = 3.4–3.62 Å, demonstrating exceptional electron mobilities of μ_e_ = 1–7 cm^2^ V^−1^ s^−1^ [2,3,10,31,35]. Figure 8 shows a representative example for π-stacking distances and CCLs through GIWAXS analysis, directly correlating structural parameters to transport pathway connectivity. In this case, despite nearly identical nanoscale packing parameters (d_π-π_ varying only 0.1 Å, CCL = 37–41 Å), electron mobility increases 12-fold from P1 (μ_e_ = 0.32 cm^2^ V^−1^ s^−1^) to P4 (μ_e_ = 3.73 cm^2^ V^−1^ s^−1^). This dramatic enhancement demonstrates that mesoscale aggregate connectivity governs transport as much as nanoscale crystallinity. P1 forms small, isolated disordered aggregates requiring frequent inter-aggregate hopping (high energetic barriers); P2–P4 develop large, interconnected crystalline domains that minimize grain boundary encounters. P4’s bimodal packing texture uniquely provides three-dimensional transport pathways, i.e., the combination of face-on (out-of-plane π-stacking) and edge-on (in-plane π-stacking) orientations creates percolation networks that circumvent microstructural defects, enabling charges to detour around grain boundaries rather than hopping across high-resistance interfaces. This structure exemplifies the design principle that transport pathway connectivity matters as critically as molecular packing distance for achieving high mobility in multi-acceptor polymers.

Why do multi-acceptor and A–A architectures pack tightly? The answer lies in their molecular electrostatic properties. Electron-deficient building blocks possess quadrupolar or multipolar charge distributions that favor co-facial π-stacking geometries. Additionally, the absence of electron-rich donor segments eliminates repulsive quadrupole-quadrupole interactions that force the polymers apart. Fluorination further enhances this effect: F···S and F···H contacts provide additional enthalpic stabilization for close-packed structures without introducing the steric penalty of larger halogens [2].

In addition to distance, the spatial extent of the ordering is significant. CCL, extracted from the width of the (010) diffraction peak via Scherrer analysis, quantifies how many π-π stacked chains maintain coherent registry before encountering defects or grain boundaries. pDFB-TF demonstrates a CCL exceeding 500 Å, corresponding to roughly 140 stacked chains in perfect registration [2]. Such extensive ordering creates well-connected transport pathways, where electrons can hop laterally across many molecules without encountering domain boundaries. In contrast, polymers with CCL < 50 Å (fewer than 15 stacked chains) suffer from frequent interruptions in the π-stacked network, forcing carriers to navigate energetically costly grain boundaries.

### 4.3. Chain Orientation and Texture

**Figure 9:**
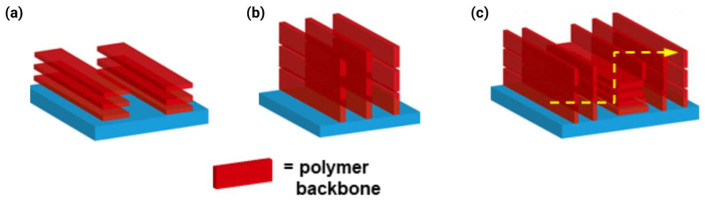
Polymer chain orientation modes. Schematic representation of (**a**) face-on (π-stacking out-of-plane), (**b**) edge-on (π-stacking in-plane), and (**c**) bimodal orientations. Bimodal packing provides three-dimensional transport pathways that circumvent grain boundaries. Reproduced with permission from Ref. [3]. Copyright 2025, American Chemical Society.

Three primary orientation modes exist: edge-on (polymer backbones perpendicular to substrate, π-stacking in-plane), face-on (backbones parallel to substrate, π-stacking out-of-plane), and bimodal (mixture of both). Figure 9 illustrates how bimodal orientation combining in-plane and out-of-plane π-stacking enables multidirectional charge transport pathways resilient to microstructural defects. For bottom-gate OFETs, edge-on orientation is traditionally considered optimal because it aligns π-stacking planes with the channel direction, maximizing lateral electronic coupling [3,36]. The A–A polymer P(NDI2OD-BiTz) achieved a decent μ_e_ of 0.11 cm^2^ V^−1^ s^−1^ specifically due to its highly ordered edge-on packing, despite predicted backbone torsion, which is a clear demonstration that favorable orientation can partially compensate for sub-optimal molecular structure [11]. Similarly, selenium-substituted polymers exhibited stronger edge-on π-stacking interactions than their sulfur analogs, correlating with superior electron transport [15].

Face-on orientation, where π-stacking runs perpendicular to the substrate, initially appears disadvantageous for lateral transport. Indeed, pure face-on packing of thiazole-based polymers contributes to their low mobility [31]. However, this view oversimplifies the three-dimensional nature of charge transport in OFETs. The accumulation layer, where the mobile charges reside, extends only 3–5 nm (approximately 8–14 polymer layers) into the film from the dielectric interface. Within this confined region, charges must navigate both laterally (for source-drain current) and vertically (to circumvent defects and grain boundaries). Pure face-on or edge-on orientations essentially constrain the transport to essentially two-dimensional pathways.

Bimodal packing textures can overcome these limitations by providing transport pathways along multiple directions. The pDFB-TF adopts a bimodal orientation with d_π-π_ = 3.62 Å in both the in-plane and out-of-plane directions [2]. When electrons encounter a grain boundary—an inevitable feature of semi-crystalline polymers—the presence of perpendicular π-stacking planes allows carriers to detour around the obstacle by hopping out-of-plane before returning to lateral transport. Several high-mobility polymers (pSNT with μ_e_ > 5 cm^2^ V^−1^ s^−1^ and P4 with short 3.40–3.45 Å stacking) share this bimodal character [3,31]. The synergy between tight π-stacking and bimodal orientation creates a robust three-dimensional transport network resilient to microstructural imperfections.

Recent work challenges the dogma that an edge-on orientation is mandatory for high mobility. Polymer P4 (Table 1), despite exhibiting significant face-on character, achieved outstanding performance due to its extraordinarily short d_π-π_ (3.40 Å)—the tightest reported for n-type polymers—enabled by additional O···H hydrogen bonding [3]. This finding reveals an important design principle: exceptionally strong electronic coupling (via ultratight stacking) can overcome unfavorable orientation geometries. The key determinant is not the orientation but rather the availability of well-connected pathways with strong electronic coupling between the chains.

The orientation is partially controlled during the film deposition. Surface energy matters; that is, hydrophobic dielectrics (HMDS-treated SiO_2_, CYTOP) tend to favor edge-on orientation by encouraging side-chain interactions with the substrate, whereas hydrophilic surfaces sometimes promote face-on packing. However, the intrinsic molecular structure, such as backbone planarity, side chain architecture, and electrostatic charge distribution, ultimately determines the orientation preferences more strongly than the processing conditions [3,31].

### 4.4. Solution Processing and Morphology Control

The transformation from dissolved polymer chains to high-performance semiconducting films represents a critical juncture at which the molecular design meets the device physics requirements. Solution processing, the cornerstone of the manufacturing promise of organic electronics, is inherently a non-equilibrium process governed by the competition between kinetic barriers (solvent evaporation rate and polymer diffusion) and thermodynamic driving forces (crystallization enthalpy and interfacial energies) [55,56]. The resulting morphology, which encompasses crystallinity, domain size, grain boundaries, and surface roughness, determines whether locally efficient transport pathways percolate to form continuous conduction channels from source to drain.

For two decades, it has been believed that high mobility requires highly crystalline large-domain microstructures. This view suggested that amorphous or poorly ordered polymers inevitably suffer from shallow trap states arising from conformational disorder, limiting mobility to <0.01 cm^2^ V^−1^ s^−1^. However, seminal work by Noriega et al. [54]. reported in 2013 fundamentally revised this understanding. By systematically studying multiple polymer families with varying degrees of crystallinity, they demonstrated that short-range intermolecular aggregation is sufficient for efficient transport and that an extended crystalline order is not necessary. The key insight is that the charge carriers in organic semiconductors have scattering mean free paths of only approximately 1–3 nm (roughly 3–8 molecules), determined by the strength of the electron-phonon coupling. Over this short distance, carriers respond primarily to local π-stacking order, not long-range crystalline registry.

**Figure 10:**
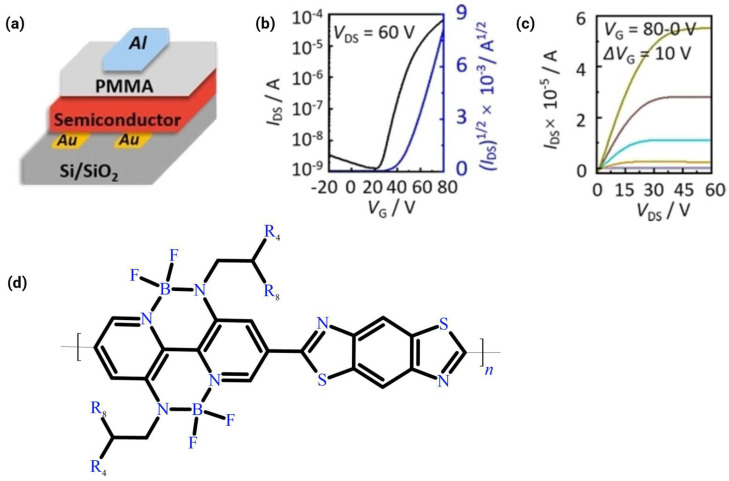
Device architecture and electrical performance of amorphous n-type polymer. (**a**) Configuration of the top-gate/bottom-contact type OFET device based on PBN-27, (**b**) transfer, and (**c**) output curves, (**d**) chemical structure of PBN-27. R_4_ = C_12_H_25_; R_8_ = C_14_H_29_. The PBN-27 demonstrates efficient transport despite poor crystallinity, validating backbone rigidity over long-range order. Reproduced with permission from Ref. [57]. Copyright 1999–2025 John Wiley & Sons, Inc.

This paradigm shifts redirected design strategies toward minimizing inherent conformational and energetic disorders rather than maximizing crystallinity. Backbone rigidity is enforced by conformational locks and fused-ring architectures, which create polymers with narrow distributions of dihedral angles and reduce site-to-site energy fluctuations, even in nominally amorphous films [4,22]. The near-amorphous n-type polymer PBN-27, despite poor crystallinity, achieved decent μ_e_ due to its highly rigid and planar backbone that maintains short-range π-overlap [57]. Figure 10 shows how the rigid backbone architecture (PBN-27) enables near-disorder-free transport in nominally amorphous films, achieving acceptable electron mobility independent of long-range crystallinity. Similarly, polymers designed with minimal backbone torsion exhibit transport approaching the disorder-free limits (Ea < 50 meV), regardless of the long-range crystalline order.

**Figure 11:**
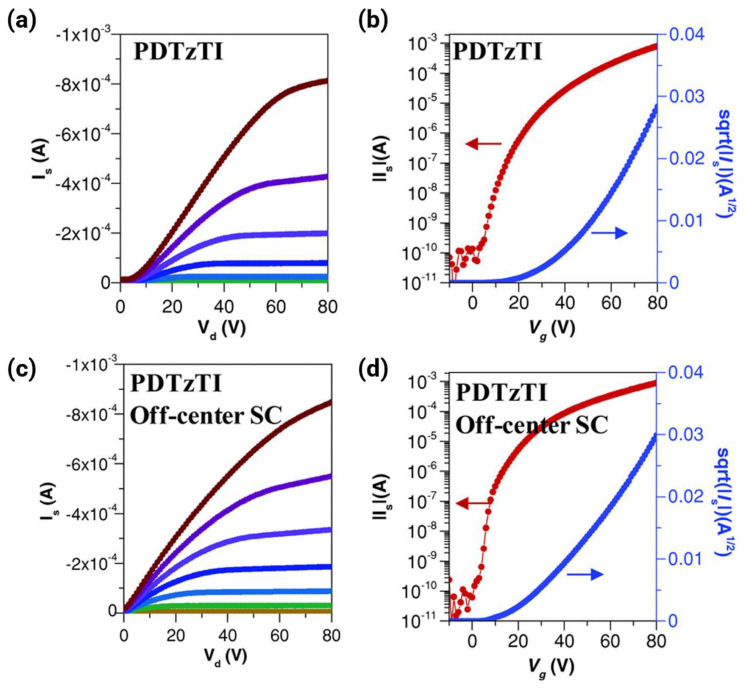
Processing-dependent device performance enhancement through directional deposition. Top-gate/bottom-contact OTFT characteristics of PDTzTI showing (**a**,**b**) on-center spin-cast devices (L = 20 µm, μₑ,max = 1.22 cm^2^ V^−1^ s^−1^, *V_T_* = 35 V) and (**c**,**d**) off-center spin-cast devices (L = 50 µm, μₑ,max = 1.61 cm^2^ V^−1^ s^−1^, *V_T_* = 24 V). Off-center deposition improves maximum electron mobility by 32% through flow-induced polymer chain alignment (dichroic ratio ~2.2) and contact resistance reduction from 112.4 kΩ·cm to 4.3 kΩ·cm. Output characteristics measured at V_d_ = 80 V showing linear and saturation regimes without S-curve distortion. Reproduced with permission from Ref. [4]. Copyright 1999–2025 John Wiley & Sons, Inc.

The most common technique employed for laboratory-scale OTFT fabrication is spin-coating [27,36,54,58]. This method involves depositing a polymer solution onto a substrate by rapidly spinning the solution to spread and evaporate the solvent, forming a uniform film, often under an inert atmosphere such as an argon-filled glovebox. The reported spin coating speeds typically range from 500 to 1500 rpm, often yielding film thicknesses of approximately 20–40 nm. High shear forces during spinning can induce partial molecular alignment, typically with limited long-range order. While spin-coating yields films with varying degrees of order, achieving macroscopic molecular alignment is a potent strategy for boosting mobility by creating quasi-one-dimensional transport pathways that minimize the number of energetically costly inter-grain hopping events. Techniques such as directional deposition (e.g., solution shearing or off-center spin coating) exploit flow-induced orientation to promote the alignment of polymer chains and crystalline domains over large areas, thereby facilitating highly efficient two-dimensional charge transport [4]. Figure 11 shows how directional deposition (off-center spin coating) improves transport by promoting polymer chain alignment and crystalline domain orientation over large areas. Off-center spin coating induces flow-driven polymer chain alignment along the source-drain direction, confirmed by polarized UV–vis absorption showing a dichroic ratio of ~2.2. This alignment creates quasi-one-dimensional transport pathways that minimize energetically costly inter-grain hopping events. Additionally, the off-center method reduces film thickness from ~65 nm to ~50 nm, decreasing contact resistance from 112.4 kΩ·cm to 4.3 kΩ·cm—more than an order of magnitude reduction.

The choice of solvent and processing conditions is instrumental in controlling the resultant thin-film morphology and crystallinity. Common solvents include chlorobenzene (CB) [17,20,58], o-dichlorobenzene (o-DCB) [28], 1,2-dichlorobenzene (DCB) [30], and chloroform [3,20,31]. Studies have demonstrated that performance is sensitive to the casting solvent through multiple mechanisms. The solvent quality (good vs. poor) affects the polymer chain conformation in solution; poor solvents promote pre-aggregation, which can seed crystallization, whereas good solvents yield more extended chains. The boiling point determines the drying kinetics; higher-boiling-point solvents such as DCB (b.p. 180 °C) provide extended time for molecular reorganization during film formation. For semi-ladder polymers, adding DCB to the solution can slow down the film-drying process, providing sufficient time for the polymers to crystallize through Ostwald ripening—the growth of larger, more perfect crystallites at the expense of smaller, defective ones—which enhanced the electron mobility significantly (e.g., improving μ_e_ to 3.13 cm^2^ V^−1^ s^−1^ in one case) [29].

Polymer solutions are typically prepared at concentrations optimized to yield high-quality films suitable for OTFT applications, frequently around 5 mg mL^−1^ or 10 mg mL^−1^ [58,59]. Solutions are usually stirred and heated (e.g., at 90 °C overnight or 120 °C for 30 min prior to spin-coating) to ensure complete dissolution and potentially promote favorable aggregation states before deposition. Heating above the aggregation temperature of the polymer ensures molecular dissolution, and controlled cooling induces the formation of ordered aggregates in the solution that serve as crystallization seeds.

Thermal annealing is a critical postdeposition process that enhances the molecular ordering and crystallinity necessary for efficient charge transport. Annealing provides thermal energy to overcome the kinetic barriers, allowing the system to explore the configurational space and approach thermodynamic equilibrium. These processes allow polymer chains to reorganize into more stable, lower-energy crystalline arrangements characterized by enhanced π-π stacking registry and increased conjugation length. The optimal annealing temperature (TA) is material-specific but typically lies within the processing window between the glass transition temperature (Tg) and the thermal decomposition temperature (Td). Observed optimal annealing temperatures are diverse: 150 °C is common for many n-type polymers [60], whereas DPP-based polymers often require 160 °C [2], and temperatures up to 220 °C or 250 °C have been used for thiazole-imide and NDI-based polymers [15,59].

To prevent the degradation of n-type polymers, which are thermodynamically susceptible to oxidation owing to their low-lying LUMO levels, thermal annealing is performed under an inert atmosphere (e.g., nitrogen- or argon-filled glovebox) to kinetically exclude oxygen. The typical duration ranged from 10 to 30 min, which was sufficient for the film to reach thermal equilibrium and complete reorganization.

**Figure 12:**
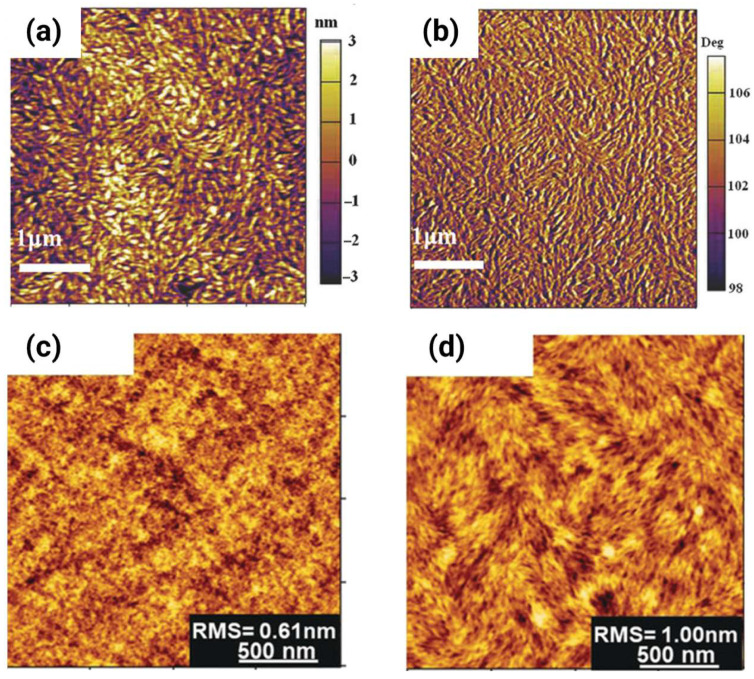
Surface morphology and charge transport correlation. (**a**) Tapping-mode AFM height and (**b**) phase images of PDTzTI films deposited on silicon substrates from chlorobenzne solution and annealed at 200 °C. Reproduced with permission from Ref. [4]. Copyright 1999–2025 John Wiley & Sons, Inc. AFM topographic height images of polymer thin films (**c**) P1 and (**d**) P2. Despite similar smoothness (both <2 nm), P2’s superior electron mobility (μ_e_ = cm^2^ V^−1^ s^−1^) demonstrates that fibrillar nanocrystalline morphology and favorable packing orientation outweigh marginal roughness differences. Reproduced with permission from Ref. [35]. Copyright 1999–2025 John Wiley & Sons, Inc.

However, crystallinity is still important when directly enhancing the properties relevant to transport. pDFB-TF’s high crystallinity (CCL = 524 Å; d_π-π_ = 3.62 Å) correlates with μ_e_ = 5.04 cm^2^ V^−1^ s^−1^ because the extended domains ensure connectivity between well-packed regions [2]. Critically, connectivity, not crystallinity, enables percolation. Films composed of isolated crystalline grains surrounded by amorphous regions may exhibit excellent local mobility within grains but poor device-level performance owing to transport bottlenecks at grain boundaries. Grain boundaries introduce energetic and structural disorders that impede the charge flow. At these interfaces, π-stacking is disrupted, creating localized trap states with binding energies of 0.1–0.3 eV. Carriers must be thermally activated over or tunnel through these barriers, degrading the effective mobility. Surface roughness exacerbates this problem: films with root-mean-square (RMS) roughness of 3–5 nm typically contain substantial grain boundaries and charge-trapping surface states. Optimal processing yields smooth films (RMS < 2 nm) with interconnected fibrillar or nanocrystalline morphologies that provide continuous pathways while maintaining local order. Figure 12 shows that interconnected fibrillar or nanocrystalline morphologies enabling continuous charge pathways matter more than minimal surface roughness variations alone.

One study demonstrated that an isomeric polymer (P2) with greater backbone torsion exhibited significantly better OTFT performance (μ_e_ up to 2.55 cm^2^ V^−1^ s^−1^) than its more planar isomer (P1) [35]. This counterintuitive result confirms that achieving highly crystalline films is often more significant than simply achieving a highly planar backbone, especially if the torsional structure facilitates favorable edge-on packing orientations and nanofiber-like surface morphologies through sterically driven self-assembly. The torsion may reduce the π-π interaction strength just enough to balance crystallization kinetics favorably, preventing kinetic trapping in metastable disordered phases.

## 5. Device Architecture and Interface Engineering

The development of high-performance n-type organic thin-film transistors utilizing multi-acceptor and A–A polymers relies fundamentally on meticulous device engineering [4,36]. While molecular design and processing optimization create intrinsic charge-transport capabilities, device architecture and interface engineering unlock this potential in functional transistors. The electrostatic field from the gate penetrates the semiconductor, creating a quasi-two-dimensional accumulation layer with a characteristic thickness of a few nanometers, where transport occurs. The choice of architecture and interfacial treatments significantly impacts the manufacturing processes, scalability, and, crucially, device performance, particularly for environmental stability.

### 5.1. Device Architecture Selection

**Figure 13:**
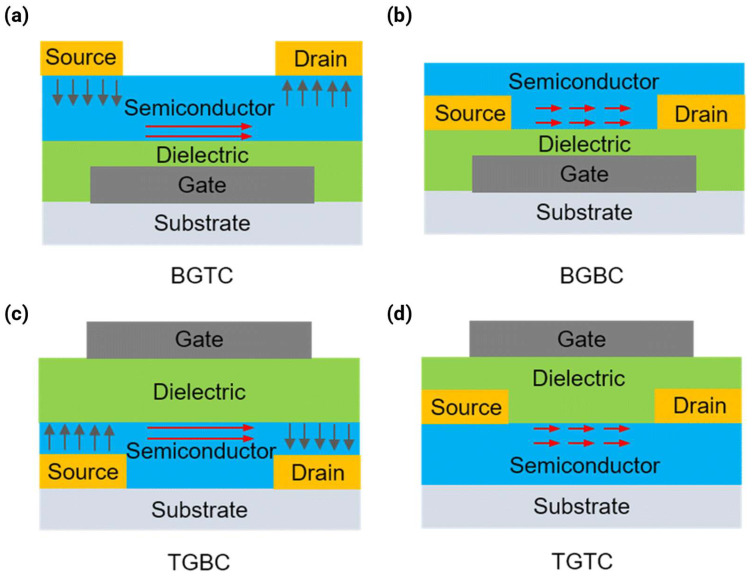
Schematic representation of four prevalent OFET device architectures distinguished by gate and source-drain electrode positioning: (**a**) bottom-gate/top-contact (BG/TC), (**b**) bottom-gate/bottom-contact (BG/BC), (**c**) top-gate/bottom-contact (TG/BC), and (**d**) top-gate/top-contact (TG/TC). Each configuration presents distinct trade-offs in terms of semiconductor-electrode interface quality, atmospheric environmental access, and practical manufacturability, which directly influence device performance and stability for n-type polymer transistors. The grey arrows represent the electric field lines and red arrows represent the direction of current flow. Reproduced with permission from Ref. [61]. Copyright 2025 Royal Society of Chemistry.

Four major device configurations are classified based on the position of the source/drain and gate electrodes: bottom-gate/top-contact (BG/TC), bottom-gate/bottom-contact (BG/BC), top-gate/bottom-contact (TG/BC), and top-gate/top-contact (TG/TC). The selection of the device architecture fundamentally determines how the semiconductor interfaces with the electrodes and the dielectric environment, thereby dictating the extent of atmospheric exposure and interface charge dynamics, as illustrated in Figure 13 for the four primary OFET geometries.

The TG/BC architecture is widely employed, especially for solution-processed polymers [2,17,20,29,32,34,58,59,60]. In this configuration, source/drain electrodes (typically gold) are patterned onto the substrate before the semiconductor film is deposited. The semiconductor layer is then covered by a dielectric layer (e.g., PMMA or CYTOP) [2,20,59], and finally, the gate electrode (e.g., aluminum) is deposited on top. In the BG/BC architecture, source/drain contacts are defined on top of the dielectric layer prior to the semiconductor deposition. This structure is advantageous for solution processing methods such as inkjet printing or spin coating, as alignment between the source/drain contacts and the gate is easily achieved. However, the interface between the semiconductor and contacts (which form the channel entry/exit points) must be managed carefully. The abrupt morphological discontinuity at the contact edges creates nucleation sites that can induce structural disorder in polymer films. This often necessitates surface treatment of the contacts or dielectric surface (e.g., using OTS-18 on the SiO_2_ surface) to promote continuous film formation and reduce contact resistance [11,36].

**Figure 14:**
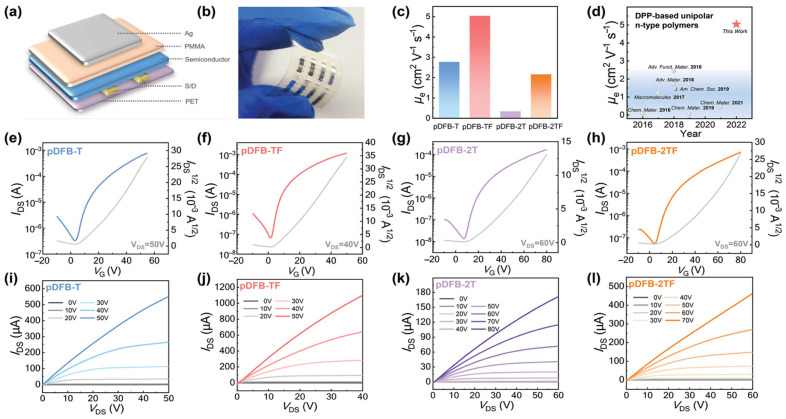
Characterization of diphenyl-fluorene (DPP)-based n-type polymer transistors fabricated in top-gate/bottom-contact geometry. (**a**) Top-gate/bottom-contact device configuration and (**b**) photograph of a flexible-transistor array on PET sheet in this work (dimension: 2 cm × 3 cm). (**c**) The electron-mobility comparison profile of the polymers. (**d**) Summary of electron-mobility values of DPP-based unipolar n-type polymers in the literature and this work. Typical n-type transfer characteristics of the transistors for (**e**) pDFB-T, (**f**) pDFB-TF, (**g**) pDFB-2T, and (**h**) pDFB-2TF. Typical n-type output characteristics of the transistors for (**i**) pDFB-T, (**j**) pDFB-TF, (**k**) pDFB-2T, and (**l**) pDFB-2TF. Reproduced with permission from Ref. [2]. Copyright 1999–2025 John Wiley & Sons, Inc.

This configuration offers several advantages to n-type polymers. By placing gate and dielectric layers on top of the semiconductor, the configuration effectively encapsulated the semiconducting layer, creating a diffusion barrier that kinetically limited the ingress of atmospheric oxygen and water molecules. This encapsulation is vital because electron-deficient polymers are thermodynamically susceptible to degradation by these species, which possess electron affinities that allow them to act as charge traps—oxygen can capture electrons to form superoxide anions (O_2_^−^), whereas water introduces hydroxyl traps that create localized states within the transport band gap. The use of a TG/BC structure generally results in a smaller hysteresis and lower V_T_ values, contributing to more reliable and stable device operation [15]. Reduced hysteresis occurs because the top dielectric deposition can passivate surface defects and dangling bonds that would otherwise act as slow-trapping sites. Figure 14 demonstrates how the TG/BC architecture delivers clean electrical signatures—steep subthreshold swings, high I_on_/I_off_ ratios, and negligible hysteresis—reflecting the quality of the encapsulated semiconductor-dielectric interface.

In contrast, the BGTC configuration allows for easy modification of the dielectric surface prior to semiconductor deposition [15,27,35]. However, because the active semiconductor layer and the critical source/drain contact region remain exposed to the ambient environment, electron-trapping atmospheric species can readily diffuse into the accumulation channel. Consequently, BG/TC devices often require measurements to be conducted in vacuum or inert atmosphere to observe the intrinsic transport properties without the confounding effects of environmental doping and trap formation. Table 2 summarizes the device architecture, dielectric engineering, contact modifications, and extracted electron mobilities alongside switching characteristics for representative high-performance n-type transistors.

### 5.2. Dielectric Material Selection and Interface Optimization

The interface between the polymeric semiconductor and the gate dielectric is paramount for charge transport, as the channel forms directly at this boundary within the Debye screening depth [7]. Several materials are utilized as gate dielectrics, each offering distinct advantages: Silicon dioxide (SiO_2_), often used in bottom-gate structures, is readily available on highly n-doped silicon substrates. However, the native surface of SiO_2_ is hydrophilic and rich in silanol (Si-OH) groups, which act as electron traps through their polar nature and ability to coordinate with π-electron systems. Additionally, the high surface energy mismatch with organic semiconductors leads to poor wetting and dewetting during solution processing, resulting in films with high defect densities and discontinuous coverage [15,27,30,36].

Polymethyl methacrylate (PMMA) is used frequently as a solution-processable dielectric layer, particularly in the TG/BC configuration [2,17,58]. Thicknesses vary, such as ≈900 nm or ≈500 nm corresponding to a capacitance (C) of 6.2 nF cm^−2^ [20]. PMMA’s amorphous structure minimizes interfacial roughness, while its carbonyl groups can weakly coordinate with polymer backbones, promoting ordered nucleation during film formation.

Fluoropolymers like CYTOP provide an ultralow surface energy, non-polar interface [2,17,20,29,33]. CYTOP layers, such as one about 400 nm thick providing a C of 4.4 nF cm^−2^, are beneficial because the absence of permanent dipoles and the chemical inertness of the C-F bonds eliminate dipolar disorder and trap states, often leading to excellent device performance approaching near-ideal characteristics. The hydrophobic nature also prevents moisture accumulation at the critical interface.

#### 5.2.1. Self-Assembled Monolayer (SAM) Engineering

SAMs are widely used to modify the dielectric surface chemistry, mediating the interfacial interactions and controlling the orientation and morphology of the overlying semiconductor film through both energetic and entropic effects. SiO_2_ surfaces are frequently functionalized with octadecyltrimethoxysilane (OTMS) [3,15]. or octadecyltrichlorosilane (OTS) [11,36]. to create a hydrophobic surface. These long-chain alkylsilanes form densely packed monolayers that effectively shield the underlying polar silanol groups, passivating trap states. The resulting low-energy surface (surface energy ~20–25 mN/m) provides better wetting for organic semiconductors cast from nonpolar solvents and promotes edge-on molecular orientation due to favorable van der Waals interactions between the alkyl chains and the polymer side chains.

**Figure 15:**
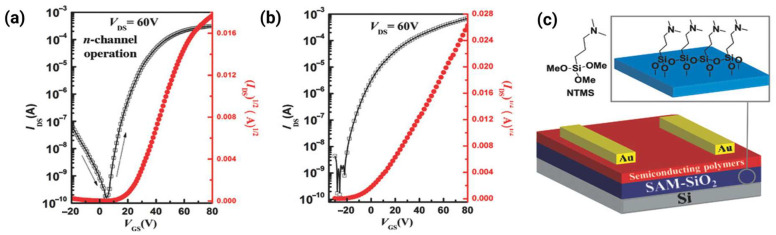
Transistor performance enhancement through amine-based surface modification. Transistor performances of transfer characteristics for pSNT (**a**) without and (**b**) with NTMS-SAM modification; (**c**) Transistors structures with the newly developed NTMS-SAM. Reproduced with permission from Ref. [31]. Copyright 1999–2025 John Wiley & Sons, Inc.

For n-channel polymers, the key goal is to enhance electron injection and suppress minority carrier (hole) injection. Amine-based SAMs have proven highly effective for this purpose through their interfacial dipole formation. For instance, [3-(N,N-dimethylamino)propyl]trimethoxysilane (NTMS), an amine-tailed SAM, was employed to modify the Si/SiO_2_ surface [31]. The amine functionalities create a strong interfacial dipole moment with the nitrogen lone pair oriented toward the semiconductor, effectively lowering the local work function and creating an electric field that attracts and stabilizes electrons near the interface. The amine-functionalized NTMS-SAM creates a strong interfacial dipole that effectively gates the charge carrier selectivity, as illustrated in Figure 15, where transfer characteristics reveal nearly complete suppression of p-type conduction following surface modification.

Using a highly smooth NTMS-SAM deposited by spin-coating led to near-perfect unipolar n-type TFTs for the polymer pSNT, yielding an electron mobility up to 5.35 cm^2^ V^−1^ s^−1^, a remarkably high current on/off ratio (≈10^7^), and a very low threshold voltage (≈1 V) [31]. This functionalization completely suppresses hole injection and transport even under p-type operating conditions. The mechanism involves the raising of the effective injection barrier for holes while simultaneously lowering it for electrons, coupled with the electrostatic gating effect that preferentially accumulates electrons.

#### 5.2.2. Contact Engineering for Efficient Charge Injection

The efficiency of charge injection from the source/drain electrodes into the semiconductor is critical for achieving high-performance OTFTs. For n-type operation, this involves aligning the Lowest Unoccupied Molecular Orbital (E) of the polymer with the metal Fermi level [31]. Gold is widely used as the electrode material due to its chemical stability and ease of deposition. However, Au typically has a high work function (Φ ~5.1 eV), making it naturally suitable for p-type injection but potentially problematic for n-type injection. For polymers with E around −3.8 eV, this barrier can exceed 1 eV, severely limiting injection efficiency.

**Figure 16:**
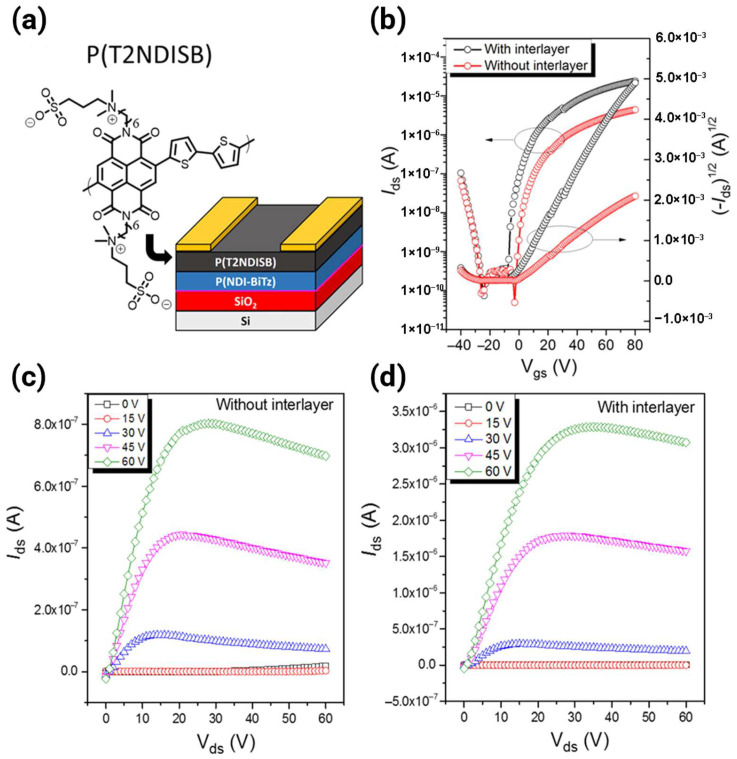
Zwitterionic polymer interlayer engineering for work function tuning. (**a**) BG/TC device architecture with the structure of P(T2NDISB) zwitterionic interlayer, (**b**) transfer characteristics of devices with and without interlayer, (**c**) output curves of devices without interlayer, and (**d**) with the interlayer. The strong, permanent dipole orientation within the zwitterionic polymer layer effectively lowers the metal Fermi level, aligning it more favorably with the polymer LUMO and reducing the electron injection barrier. Reproduced with permission from Ref. [11]. Copyright 2025, American Chemical Society.

When the electrode work function is not ideally matched, strategies are employed to minimize the contact resistance (R). Inserting thin interfacial layers between the metal electrode and the semiconductor can modify the effective work function through interfacial dipole formation. CsF has been utilized, typically spin-cast onto the Au source/drain electrodes in TG/BC architectures. Upon contact with the organic semiconductor, CsF dissociates and the highly electropositive Cs atoms donate electron density to the metal, creating a large interfacial dipole that lowers the work function by 1–2 eV, dramatically reducing the electron injection barrier [28,29]. Similarly, an ultrathin PEIE layer has been successfully incorporated between Au electrodes and the semiconductor layer [34]. The amine groups in PEIE create a dipole layer similar to NTMS but at the contact interface, lowering the local work function and facilitating electron tunneling through any residual barrier. In addition, Zwitterionic polymer interlayer was also reported for lowering the electrode work function. Zwitterionic materials contain both cationic and anionic groups within the same molecule, creating strong, permanent dipoles. Materials such as P(T2NDISB) have been investigated as interlayers in BG/TC devices, where their dipole orientation can be controlled during deposition to favorably shift energy levels [11]. Figure 16 demonstrates the device performance enhancement after the inclusion of P(T2NDISB) interlayer, between the channel and the source/drain contact electrodes.

The inherent material properties, particularly microstructure and chain organization, also play a key role in minimizing R by enabling efficient charge extraction even in the presence of modest injection barriers. Polymers with robust, ladder-type structures or conformationally locked backbones exhibit substantially enhanced electron transport and display a dramatically reduced source/drain contact resistance. For instance, modeling parameters derived from AIM-SPICE analysis showed that BBL had an source/drain resistance of 0.9 × 10^6^ Ω, significantly lower than the 15 × 10^6^ Ω observed for the semi-ladder BBB [27]. This reduction in R arises because the ladder structure enforces complete backbone planarity and maximizes π-orbital overlap along the chain, creating extended conjugation lengths. The resulting delocalized electronic states couple more efficiently with metal electrodes and reduce sensitivity to local disorder at the interface.

### 5.3. Performance Metrics and Optimization

The performance of organic thin-film transistors based on multi-acceptor and all-acceptor polymers must be rigorously quantified and evaluated using standardized metrics to enable accurate comparisons and guide materials development. Device analysis is complex due to the inherent disorder and variability of organic semiconductors, necessitating robust extraction techniques to ensure the reported charge transport values reflect the material’s intrinsic potential rather than device artifacts.

The field-effect mobility (μ) stands as the quintessential figure-of-merit, characterizing the efficiency of charge transport within the semiconducting channel. Charge transport in conjugated polymers is often described using various models—band-like transport, hopping, or multiple trapping and release—reflecting the material’s intermediate position on the order-disorder scale [54]. The transport mechanism depends on the degree of electronic delocalization, with highly ordered systems exhibiting quasi-band-like transport (characterized by negative temperature coefficient of mobility) and disordered systems showing thermally activated hopping (positive temperature coefficient). Regardless of the underlying microscopic mechanism, mobility is typically extracted macroscopically from the device electrical characteristics using the standard MOSFET equations derived from the gradual channel approximation model [27].

#### 5.3.1. Ensuring Reliability in Mobility Extraction

High-performance polymers should exhibit “kink-free” transfer characteristics and minimal drain current hysteresis in the I versus V plots, indicating negligible slow trap states [29,54,62]. The extracted mobility values should show weak dependence on the gate voltage (V) above the threshold. If the mobility rises significantly upon increasing the magnitude of V, it typically indicates trap-dominated transport where increasing gate voltage progressively fills deeper traps. Some V-dependent transport is frequently observed in high-mobility semicrystalline polymers, attributed to intrinsic carrier concentration effects like Coulomb enhanced transport where increased carrier density screens disorder. However, a weak dependence—mobility varying by less than factor of 2–3 over the operating range—is ideal and indicates that trap density is low [10].

For high reliability, the electron mobilities extracted from the saturation (μ) and linear (μ) regimes should be nearly the same, ideally within 20–30%. Significant discrepancy suggests contact-limited transport or non-ideal behavior. For example, in one study, the maximum saturation and linear electron mobilities were reported as 0.72 and 0.67 cm^2^ V^−1^ s^−1^, respectively, indicating high reliability and negligible contact resistance contribution [38].

**Figure 17:**
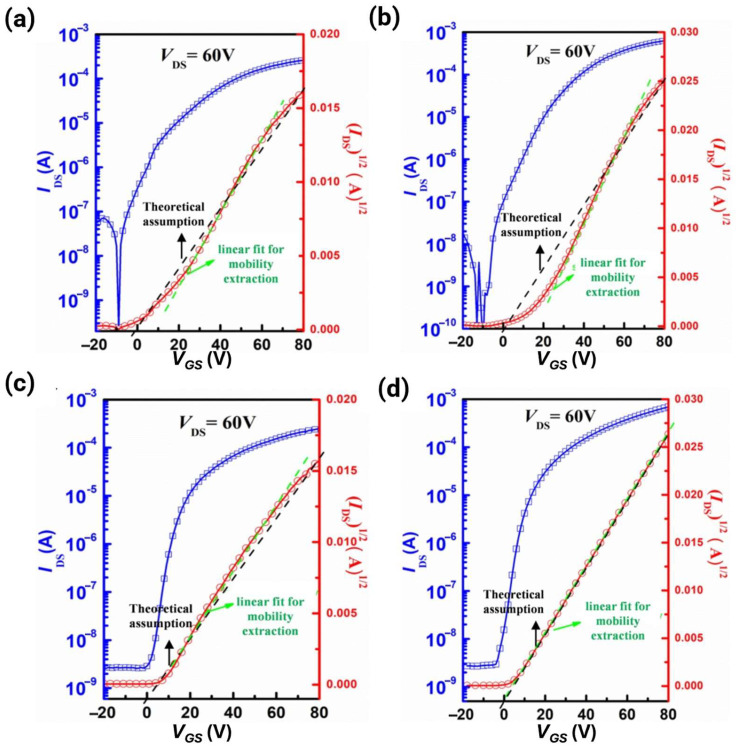
Device quality assessment via reliability factor (γ). The measurement reliability factor (γ) is the ratio, expressed in %, of the slopes of the black (theoretical assumption) and green dashed lines (linear fit for mobility extraction) for the OTMS-treated PTFTs based on (**a**) P3 (γ ≈ 85%); (**b**) P4 (γ ≈ 80%); and the NTMS-treated PTFTs based on (**c**) P3 (γ ≈ 92%); (**d**) P4 (γ ≈ 100%). The progression demonstrates that NTMS surface modification improves device ideality, with P4/NTMS approaching perfect square-law behavior. Reproduced with permission from Ref. [3]. Copyright 2025, American Chemical Society.

A more quantitative metric for reliability is the factor γ, proposed to gauge the reliability of reported values. The factor γ is essentially the ratio of the experimentally derived slope to the theoretically ideal slope expected from the MOSFET equation assuming perfect square-law behavior [3]. Values for γ lower than 100% signify mobility over-estimation due to non-idealities such as contact resistance, trap filling, or gate-voltage-dependent mobility. High-quality devices should exhibit reliability factors exceeding 75%, with ideal devices approaching γ ≈ 100%. Notably, in certain n-type polymers utilizing advanced surface modification (NTMS-SAM), γ values approached 100% (shown in Figure 17), underscoring the high reliability of the extracted mobility and near-ideal transistor behavior [3].

#### 5.3.2. Molecular Weight and Processing Optimization

The relationship between the polymer’s molecular weight (MW or M) and its charge transport properties is a critical but complex design guideline rooted in multiple competing factors. Studies show that increasing the MW or degree of polymerization of semiconducting polymers can lead to improvements in photoresponsivity, charge transport, and interfacial ordering [31,32,54]. Longer chains provide greater inter-chain entanglement and physical cross-linking, improving mechanical integrity and facilitating tie-chain mediated charge transport between crystalline domains. However, this improvement typically plateaus after a certain chain length (often DP ~ 50–100 repeat units), often due to paracrystallinity reaching a limit—beyond a critical length, the entropic penalty for perfect chain alignment in crystals becomes prohibitive, and the defect density saturates.

Techniques like optimized Stille polymerization protocols can successfully yield high-quality n-type polymers with number-average molecular weights up to 10^5^ g mol^−1^ [31]. For semi-ladder polymers, Pd-catalyzed Stille coupling proved superior to Ni-mediated Yamamoto coupling in producing polymers with higher MW and quality, correlating to increased μ [29]. The superior performance of Stille coupling likely stems from its higher functional group tolerance and better control over stoichiometry, reducing chain-end defects.

For all-acceptor polymers, a primary challenge is that high molecular aggregation affinity driven by strong π-π interactions between electron-deficient aromatic units often diminishes solubility, thereby limiting the achievable degree of polymerization [30]. This is fundamentally a solubility-crystallinity trade-off. Furthermore, while larger ladder-type building blocks increase backbone stiffness and reduce conformational entropy—beneficial for reducing energetic disorder—the extension of these units can negatively affect molecular motion during film formation. The increased persistence length reduces chain flexibility needed for defect annealing, potentially leading to increased disordered phases and a drop in mobility if processing conditions are not carefully optimized [29].

#### 5.3.3. Strategic Processing Guidelines

High-performance n-type transistor fabrication relies on synergistic optimization across material chemistry and processing physics. Efficient charge transport requires minimization of energetic and conformational disorder, dictated by molecular packing and degree of crystalline order. High performance has been achieved in systems that approach the intrinsic disorder-free limits by utilizing molecular design guidelines that enforce a planar, torsion-free backbone conformation [62]. Planar backbones maximize π-orbital overlap and conjugation length, narrowing the density of states and increasing charge delocalization, which manifests macroscopically as reduced activation energy for transport.

Ladder-type backbones, like those studied in BBL systems, and noncovalently fused-ring polymers aim to reinforce backbone stiffness and electronic delocalization by covalently or supramolecularly locking the backbone into a planar geometry [22,27,37]. Introducing vinyl bridges can also significantly enhance coplanarity and crystallinity through induced multiple intra-/intermolecular non-covalent interactions (C-H···N, C-H···π), which act as conformational locks.

Ultimately, the morphological ideal for n-type OFETs comprises: (1) sufficient short-range π-aggregation to enable efficient local hopping (d < 3.7 Å maintained over 5–10 molecules), (2) connected transport pathways that percolate across the film (either through extensive crystalline domains or well-connected nanocrystalline networks), and (3) smooth interfaces (RMS < 2 nm) that minimize grain boundary trapping. Multi-acceptor and all-acceptor polymers approach this ideal by combining strong π-stacking propensity from their electron-deficient character with backbone rigidity from conformational locks, yielding microstructures that balance order and connectivity.

### 5.4. Device Stability

Operational stability is a non-negotiable metric for the commercial viability of n-type organic thin-film transistors (OTFTs), especially those leveraging multi-acceptor and all-acceptor polymers designed for high performance. While the mobility dictates speed and current density, stability encompasses resistance to ambient species, electrical stress, and thermal fluctuations which determines the device’s functional lifetime and reliability in real-world applications.

#### 5.4.1. Ambient Stability: Time-Dependent Degradation

N-type polymers, which rely on accumulating electrons in the transport channel, are inherently susceptible to environmental degradation. The performance of these materials suffers significantly when exposed to atmospheric oxygen (O_2_) and moisture (H_2_O) [3,15,30]. These ambient species act as deep electronic traps at the semiconductor surface and within the thin film through multiple mechanisms: oxygen can undergo reduction to form superoxide radical anions (O_2_^−^ + e^−^ → O_2_^2−^) that bind to the polymer backbone, while water molecules create polar trapping sites through dipole-charge interactions and can hydrolyze reactive functional groups. Both processes capture free electrons and drastically reduce the effective carrier concentration and mobility.

The core strategy to achieve ambient electron transport stability is the chemical design of polymers possessing sufficiently deep Lowest Unoccupied Molecular Orbital (*E_LUMO_*) energy levels [20,63]. The thermodynamic stability against oxidation is governed by the energy difference between *E_LUMO_* and the O_2_/O_2_^−^ redox potential (approximately −4.0 to −4.3 eV vs. vacuum, depending on environment): when *E_LUMO_* > −4.0 eV, electron transfer to oxygen becomes thermodynamically favorable (Δ*G* < 0), leading to rapid doping and trap formation.

In addition to chemical design, device architecture provides a practical solution to environmental vulnerability. The top-gate/bottom-contact (TG/BC) configuration is often employed because the dielectric layer (e.g., PMMA) inherently acts as an encapsulant, minimizing the entry of O_2_ and H_2_O into the conduction channel by creating a diffusion barrier. The permeability of oxygen through PMMA is typically ~10^−14^ cm^2^/s, several orders of magnitude lower than diffusion through air, thereby allowing the observation of “pristine” performance shortly after fabrication [10,15]. Certain ladder-type polymers, such as the semi-ladder BBB and the full ladder BBL, have demonstrated remarkable long-term ruggedness in bottom-gate/top-contact (BG/TC) devices, maintaining stable mobility and *V_T_* for over 6 months in ambient air, although both showed an initial decrease in mobility when moved from inert conditions to air [27]. The initial drop reflects fast surface oxidation, while the subsequent stability indicates that oxidation saturates at the surface without penetrating the bulk film, likely due to the dense molecular packing in ladder structures.

#### 5.4.2. Bias Stress Stability: Interface Charge Trapping

Bias stress stability quantifies the change in device characteristics, notably the threshold voltage (*V_T_*) shift when the transistor is subjected to continuous operational electrical stress [15]. This degradation mechanism is primarily rooted in the presence of electronic traps at the critical semiconductor/dielectric interface, which capture accumulating charge carriers over time [54]. Under prolonged gate bias, mobile charges can become trapped in localized states (either in the semiconductor band tail or in the dielectric), creating a sheet of fixed charge that screens the gate field. This trapped charge must be compensated by increased gate voltage to maintain the same mobile carrier density, manifesting as a positive shift in *V_T_* for n-type devices.

The molecular structure plays a critical role in minimizing these traps. Structural disorder in semicrystalline polymers leads to electronic traps (sometimes described as gap states or band tails arising from variations in conjugation length and orbital overlap) that ultimately limit charge transport. The density of states tails can be approximated by an exponential distribution with characteristic energy E_urbach_, and the high E_urbach_ indicates high disorder and numerous shallow traps. Therefore, polymers designed to approach intrinsic disorder-free limits, typically achieved through rigid, planar, torsion-free backbone conformations, in order to exhibit superior bias stress stability by minimizing the density of these tail states.

**Figure 18:**
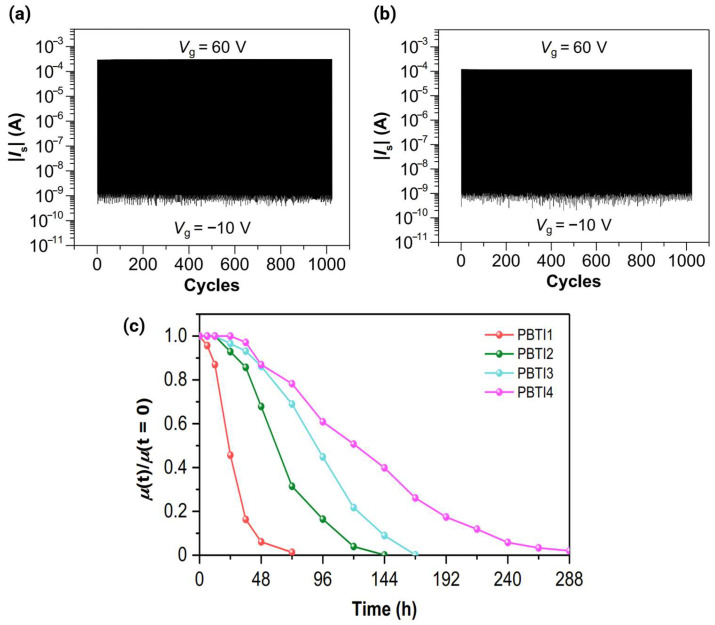
OTFT bias stability test results of homopolymers PBTIn: (**a**) PBTI1 (μₑ > 1.2 cm^2^ V^−1^ s^−1^), (**b**) PBTI2 (μₑ > 0.96 cm^2^ V^−1^ s^−1^). The devices were turned on and off for 1024 cycles in nitrogen-filled glove box; (**c**) Temporal evolution of OTFT performance of PBTIn homopolymers. The devices were stored and measured in ambient environment with a relativity humidity around 50%. Reproduced with permission from Ref. [29]. Copyright 2025, American Chemical Society.

For high-performance n-type systems, exceptional bias stress stability has been demonstrated:

- An all-acceptor polymer (P3) showed remarkable bias stress stability tested over a timescale up to 10^3^ s under continuous operation, with *V_T_* shift < 1 V, and its source-drain current (*I_DS_*) remained relatively constant (<10% decrease) after 100 on-off switching cycles in ambient conditions [3].
- The OTFTs based on PBBT-NDI showed relatively stable √*I_DS_*—VGS characteristics over 10^3^ s under bias stress with minimal hysteresis [15].
- Ladder-type BTI homopolymers exhibited negligible performance variation (*V_T_* shift < 0.5 V, mobility change < 5%) when subjected to 1024 on-off cycles (displayed in Figure 18, with temporal evolution profile), demonstrating excellent immunity to charge trapping [29].

#### 5.4.3. Temperature-Dependent Operation and Thermal Stability

The thermal stability of the polymer itself dictates the maximum permissible temperature for processing (e.g., thermal annealing) and determines the stability of the device morphology during operation. Multi-acceptor and all-acceptor conjugated polymers generally possess high thermal decomposition temperatures (T_d_), often exceeding 350 °C as measured by thermogravimetric analysis (TGA, typically defined as 5% weight loss) [32]. For a series of cyano-functionalized imide polymers, T_d_ values ranged from 310 to 350 °C [17]. This wide thermal processing window is crucial for optimizing crystallinity through thermal annealing without inducing chemical degradation [31]. The high T_d_ in these systems arises from the strong covalent bonding in the aromatic backbone and the electron-deficient nature of the acceptor units, which stabilize against thermally induced radical formation.

**Figure 19:**
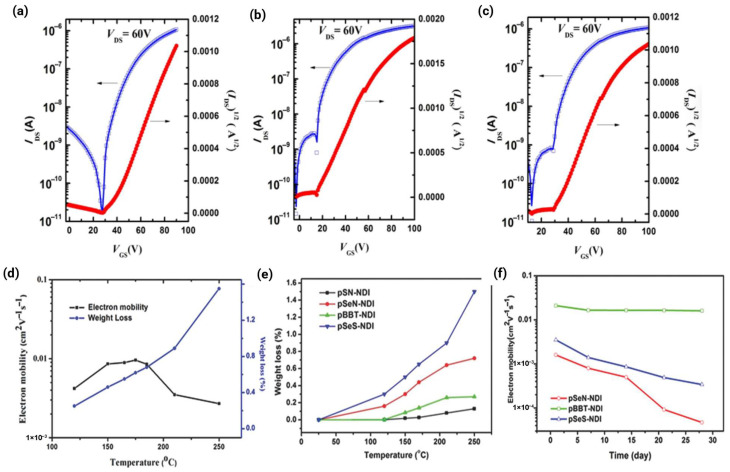
Thermal stability mismatch in device versus bulk. I–V characteristics of OTFTs based on pSeS-NDI after thermal annealing at (**a**) 150 °C, (**b**) 175 °C, and (**c**) 200 °C under vacuum of 10^−4^ mbar; (**d**) relationship between the thermal annealing temperatures and electron mobilities of pSeS-NDI; (**e**) weight loss behavior of the four polymers upon heating from 25 to 250 °C; (**f**) time-dependent changes in the electron mobilities in air. Reproduced with permission from Ref. [15]. Copyright 1999–2025 John Wiley & Sons, Inc.

However, high intrinsic thermal stability does not guarantee optimized device performance at high annealing temperatures. For instance, in the selenadiazole-based polymer pSeS-NDI, thermal annealing at 250 °C was detrimental, leading to a dramatic decrease in OTFT performance and film crystallinity, suggesting partial decomposition or structural rearrangement occurred above 175 °C despite TGA indicating stability to higher temperatures (shown in Figure 19) [15].

This underscores that the polymer’s functional stability in the device environment, where thin film confinement effects, substrate interactions, and atmospheric exposure can accelerate degradation pathways, may be significantly lower than its decomposition temperature defined solely by bulk TGA measurements. The discrepancy may arise from surface-initiated decomposition or catalytic effects from the substrate that are not captured in TGA powder measurements.

## 6. Opportunities and Challenges

### 6.1. Emerging Opportunities

Complementary Circuit Integration: All-acceptor polymers with μₑ > 5 cm^2^ V^−1^ s^−1^ now match p-type polymer performance, enabling true CMOS architectures [3,31]. Recent demonstrations of complementary inverters using pDFB-TF/P3HT pairs achieved voltage gains of 44 *v*/*v* at 3 V operation—approaching silicon CMOS benchmarks [5]. The key opportunity lies in developing matched n-type/p-type pairs with identical processing conditions to simplify manufacturing.

Bioelectronics Applications: The deep LUMO levels (−4.0 to −4.4 eV) that ensure ambient stability also enable operation in aqueous environments. Organic electrochemical transistors (OECTs) using all-acceptor polymers demonstrate transconductance exceeding 15 mS with response times below 1 ms—suitable for neural recording [5,64]. The cyano-functionalized polymers show particular promise due to their mixed ionic–electronic conduction.

Thermoelectric Devices: N-type doping of all-acceptor polymers using hydride donors (N-DMBI) achieves electrical conductivities above 10 S cm^−1^ while maintaining Seebeck coefficients above −200 μV K^−1^. The rigid backbone structures minimize phonon transport, yielding power factors up to 53.4 μW m^−1^ K^−2^ and ZT values of 0.01 at room temperature [65]. Integration with existing p-type organic thermoelectrics could enable flexible energy harvesters operating from body heat.

### 6.2. Persistent Challenges

Molecular Weight Limitations: All-acceptor polymerization typically yields Mₙ < 50 kDa due to precipitation during synthesis. While sufficient for transistors, applications requiring mechanical flexibility need Mₙ > 100 kDa. Current strategies using mixed-solvent systems only partially address this limitation.

Batch-to-Batch Variability: Device performance varies by ±30% between polymer batches even with identical synthesis protocols. Multiple factors contribute to this irreproducibility: (i) trace palladium catalyst residues (>10 ppm) act as charge traps that degrade mobility, (ii) end-group composition (H-terminated vs. Br-terminated chains) affects injection barriers and creates interface dipoles, and (iii) subtle molecular weight variations alter film morphology and tie-chain connectivity between crystalline domains. Addressing these issues requires implementing a comprehensive analytical protocol: inductively coupled plasma mass spectrometry (ICP-MS) for quantifying Pd residues below 1 ppm detection limits, matrix-assisted laser desorption/ionization time-of-flight mass spectrometry (MALDI-TOF MS) for end-group identification, and size-exclusion chromatography with multi-angle light scattering (SEC-MALS) for absolute molecular weight determination independent of polymer architecture. Together, these techniques provide the analytical rigor needed to correlate synthesis variables with device performance and establish meaningful batch-release specifications.

Environmental Degradation Mechanisms: While ambient stability improved dramatically, devices still degrade under UV exposure and elevated humidity (>80% RH). Photo-oxidation creates carbonyl defects along the backbone, introducing trap states. Encapsulation adds cost and complexity, particularly for flexible substrates where barrier layers must withstand mechanical strain.

Contact Resistance Scaling: As channel lengths approach 1 μm for high-frequency applications, contact resistance dominates total resistance even with work-function-modified electrodes. The width-normalized contact resistance (RcW) of 100–500 Ω·cm limits practical device dimensions. Developing molecular dopants for selective contact doping remains an open challenge.

Processing-Performance Trade-offs: High-performance polymers require chlorinated solvents (chlorobenzene, o-dichlorobenzene) incompatible with industrial safety standards. Green solvent alternatives (toluene, p-xylene) yield films with 50–70% lower mobility due to inadequate polymer dissolution and rapid crystallization kinetics. Developing polymers specifically designed for non-halogenated processing represents a critical need.

## 7. Summary and Outlook

Multi-acceptor and all-acceptor polymer architectures have emerged as the dominant strategy for achieving unipolar n-type organic semiconductors. The systematic progression from dual-acceptor through triple-acceptor to all-acceptor designs demonstrates clear structure-property relationships: each additional acceptor unit deepens both LUMO and HOMO levels by approximately 0.1–0.2 eV while maintaining the backbone planarity essential for efficient charge transport.

Current achievements include electron mobilities exceeding 7 cm^2^ V^−1^ s^−1^ (polymer P4 with NTMS-SAM treatment), on/off ratios reaching 10^7^–10^8^ (PDTzTI), and threshold voltages reduced to 1–5 V through amine-functionalized SAMs. The triple-acceptor polymer pDFB-TF exemplifies optimized performance with μₑ = 5.04 cm^2^ V^−1^ s^−1^, crystalline coherence length of 524 Å, and months-long ambient stability—demonstrating that fluorination-induced conformational locking successfully addresses both electronic and microstructural requirements simultaneously.

Critical insights from transport studies reveal that short-range π-aggregation (5–10 molecules) with tight stacking distances (3.4–3.6 Å) matters more than long-range crystallinity. This finding redirects synthetic efforts from pursuing high crystallinity toward minimizing conformational disorder through backbone rigidification. The bimodal packing orientation observed in high-performance systems provides three-dimensional transport networks that circumvent grain boundaries—a previously underappreciated design element.

Three technical challenges limit immediate commercialization. First, the inverse relationship between stability (requiring LUMO < −4.0 eV) and operating voltage (favoring shallower LUMOs) remains unresolved. Second, synthetic yields for all-acceptor polymers rarely exceed 60% due to the low reactivity of electron-deficient monomers, despite advances in direct arylation protocols. Third, contact resistance still accounts for 30–50% of total device resistance even with optimized interfaces, masking intrinsic transport capabilities.

Near-term developments should focus on: (i) developing new acceptor scaffolds combining benzothiadiazole-type electron deficiency with reduced steric hindrance, (ii) implementing in-situ polymerization techniques to overcome solubility limitations while achieving higher molecular weights, and (iii) establishing standardized testing protocols including humidity-temperature cycling to predict operational lifetimes. The convergence of molecular design principles, refined processing methods, and interface engineering positions multi-acceptor polymers to enable complementary circuits operating below 5 V with carrier mobilities approaching 10 cm^2^ V^−1^ s^−1^—the threshold for commercial organic electronics.

## Figures and Tables

**Table 1:** Molecular and Synthetic Properties of Multi-Acceptor and All-Acceptor Polymers.

Polymer Designation (or Acronym Used in Literature)	Acceptor Building Block(s)	LUMO/HOMO Levels	Synthesis Method	Molecular Weight (Mn/Mw) (kg/mol)	Ref
pDBF-T DPP-Difluorobenzothiadiazole (DFB) triad copolymer	Diketopyrrolopyrrole (DPP), Difluorobenzothiadiazole (DFB) triad	LUMO: −3.60 eV; HOMO: −5.62 eV	Pd-catalyzed Stille-coupling polymerization	Mn: 33.9/Mw: N/A	[2]
pDBF-TF DPP-DFB triad copolymer with difluorothiophene donor	Diketopyrrolopyrrole (DPP), Difluorobenzothiadiazole (DFB) triad	LUMO: −3.74 eV; HOMO: −5.66 eV	Pd-catalyzed Stille-coupling polymerization	Mn: 34.4/Mw: N/A	[2]
pDBF-2TF DPP-DFB triad copolymer with difluorobithiophene donor	Diketopyrrolopyrrole (DPP), Difluorobenzothiadiazole (DFB) triad	LUMO: −3.62 eV; HOMO: −5.62 eV	Pd-catalyzed Stille-coupling polymerization	Mn: 53.2/Mw: N/A	[2]
P3 (NDI/BBTV/Vinylene) NDI/BBTV-F/Vinylene Copolymer	Naphthalene diimide (NDI), Fluorinated vinylene-bridged bis(benzothiadiazole) (BBTV-F), Vinylene spacer	LUMO: −3.80 eV; HOMO: −5.71 eV	Pd(0)/CuI co-catalyzed Stille coupling polycondensation	Mn: 31.6/PDI: 2.5	[3]
P4 (NDI/BBTV/Vinylene + SN) Vinylene-substituted pSNT analog	Naphthalene diimide (NDI), Thiadiazolebenzotriazole (SN), Vinylene spacer	LUMO: −3.87 eV; HOMO: −5.40 eV	Pd(0)/CuI co-catalyzed Stille coupling polycondensation	Mn: 54.9/PDI: 1.8	[3]
PDTzTI Poly(2,2′-bithiazolothienyl-4,4′,10,10′-tetracarboxydiimide)	Bithiazolothienyl-tetracarboxydiimide (DTzTI)	LUMO: −3.77 eV; HOMO: −5.78 eV	Pd-catalyzed Stille-coupling polymerization (CuI cocatalyst used for monomer BTzI)	N/A	[4,20]
P2 (NBA/TVT) NBA/TVT copolymer (NBA2DH-TVT)	Naphthalene bis(4,8-diamino-1,5-dicarboxyl)amide (NBA), 1,2-di(2-thienyl)ethylene (TVT)	LUMO: −3.50 eV; HOMO: −5.51 eV	Pd-catalyzed Stille-coupling polymerization	Mn: 38.1/Mw: 143.7	[10]
P3 (NBA/DTBT) NBA/DTBT copolymer (NBA2DH-DTBT)	Naphthalene bis(4,8-diamino-1,5-dicarboxyl)amide (NBA), 4,7-di(2-thienyl)-2,1,3-benzothiadiazole (DTBT)	LUMO: −3.57 eV; HOMO: −5.57 eV	Pd-catalyzed Stille-coupling polymerization	Mn: 61.8/Mw: 225.6	[10]
P4 (NBA/DTBTff) NBA/DTBTff copolymer	Naphthalene bis(4,8-diamino-1,5-dicarboxyl)amide (NBA), 5,6-difluoro-4,7-di(2-thienyl)-2,1,3-benzothiadiazole (DTBTff)	LUMO: −3.59 eV; HOMO: −5.92 eV	Pd-catalyzed Stille-coupling polymerization	Mn: 108.0/Mw: 625.7	[10]
BDPPV Benzodifurandione-based PPV	Benzodifurandione-based oligo(p-phenylene vinylene) (BDOPV) derived unit	LUMO: −4.10 eV; HOMO: −6.12 eV	Stille coupling polymerization using (E)-1,2-bis(tributylstannyl)ethene	Mn: 37.6/PDI: 2.38	[18]
P2N2F-4FBT Bisisoindigo/Tetrafluorobithiophene Copolymer (Azaisoindigo derivative)	Bisisoindigo (bis-azaisoindigo derivative) containing 2 N and 4 F atoms	LUMO: −4.01 eV; HOMO: −6.04 eV	Microwave assisted Stille coupling polycondensation	Mn: 20.3/PDI: 2.20	[21]
PBTI1 Poly(bithiophene imide 1) (Semi-ladder type)	Bithiophene imide (BTI1)	LUMO: −3.48 eV; HOMO: −5.46 eV	Pd-catalyzed Stille coupling	Mn: 12.7/PDI 2.1	[29]
pPTT NDI copolymer with Benzothiadiazole derivative	Naphthalene diimide (NDI) and Benzothiadiazole (BT) derivative containing one sp^2^-N atom	LUMO: −3.81 eV; HOMO: −5.87 eV	Improved Stille cross-coupling polymerization (Pd(0)/Cu(I) cocatalyst, high Mn protocol)	Mn: 73.2/Mw: 146.3	[31]
pSNT NDI copolymer with Thiadiazolebenzotriazole (SN)	Naphthalene diimide (NDI) and Thiadiazolebenzotriazole (SN) (triple-fused-ring structure)	LUMO: −3.88 eV; HOMO: −5.45 eV	Improved Stille cross-coupling polymerization (Pd(0)/Cu(I) cocatalyst, high Mn protocol)	Mn: 61.3/Mw: 154.2	[31]
PBTI Poly(bithiophene imide) (Synthesized from distannylated monomer)	Bithiophene imide (BTI)	LUMO: −3.45 eV; HOMO: −5.43 eV	Pd-catalyzed Stille coupling of distannylated BTI-Tin with dibrominated BTI-Br	Mn: 35.5/PDI: 1.7	[32]
P(BTI-BTI2) Bithiophene imide copolymerized with fused BTI2	Bithiophene imide (BTI), Fused Bithiophene Imide (BTI2)	LUMO: −3.55 eV; HOMO: −5.43 eV	Pd-catalyzed Stille coupling of distannylated BTI-Tin with dibrominated BTI2-Br	Mn: 39.4/PDI: 3.0	[32]
PBTI* Poly(bithiophene imide) (Synthesized from dibrominated monomer)	Bithiophene imide (BTI)	LUMO: −3.48 eV; HOMO: −5.46 eV	Pd-catalyzed Stille coupling of dibrominated monomer (BTI-Br)	Mn: 12.7/PDI: 2.1	[32]
PBTI2* Poly(bithiophene imide 2) (fused structure from dibrominated monomer)	Fused Bithiophene Imide (BTI2)	LUMO: −3.53 eV; HOMO: −5.39 eV	Pd-catalyzed Stille coupling of dibrominated monomer (BTI2-Br)	Mn: 13.5/PDI: 2.2	[32]
PCNDFDE-DPP Poly(2,3-di-fluoro-1,4-dicyano-butadiene-alt-DPP)	Diketopyrrolopyrrole (DPP), 2,3-di-fluoro-1,4-dicyano-butadiene (CNDFDE)	LUMO: −4.06 eV; HOMO: −5.79 eV	Pd-catalyzed Stille-coupling polymerization	Mn: 55.6/PDI: 2.7	[33]
P2 (NDI/Thiazole Isomer 2, vacuum) Isomeric NDI/Thiazole All-Acceptor Polymer (Isomer 2)	Naphthalene diimide (NDI), Thiazole derivative (positional isomer)	LUMO: −4.00 eV; HOMO: −6.15 eV	Direct Arylation Polycondensation (DArP) using Pd/Cu co-catalysts (modified method)	Mn: 58.4/PDI: 2.1	[35]
DPPTh-4ClTVT Diketopyrrolopyrrole/Chlorinated TVT Copolymer	Diketopyrrolopyrrole (DPP), (E)-1,2-bis(3,4-dichlorothien-2-yl)ethene (4ClTVT)	LUMO: −3.58 eV; HOMO: −5.41 eV	Direct Arylation Polycondensation (DArP)	Mn: 70.6/PDI: 2.0	[34]

PBTI* and PBTI2* synthesized from dibrominated monomers BTI-Br and BTI2-Br, used for comparison from the literature.

**Table 2:** Device Performance Metrics and Transport Characteristics of Multi-Acceptor and All-acceptor n-Type PFETs.

Polymer/Device Code	Device Architecture	Dielectric Used (Material & Thickness)	Contact Modification (Electrode Material, Geometry)	Saturation/Average Electron Mobility (µ_e_, Max/µ_e_, Avg) cm^2^ V^−1^ s^−1^	On-Off Ratio/OFF Current/Threshold Voltage (I_on_/I_off_, V_T_ V)	Ref.
pDBF-T	TG/BC	PMMA	Gate—Ag; Source & Drain—Cr/Au	max: 2.77; avg: 2.29	10^3^–10^5^; 15.5	[2]
pDBF-TF	TG/BC	PMMA	Gate—Ag; Source & Drain—Cr/Au	max: 5.04, avg: 3.61	≈10^5^; 14.1	[2]
pDBF-2TF	TG/BC	PMMA	Gate—Ag; Source & Drain—Cr/Au	max: 2.16, avg: 1.35	≈10^4^; 27.8	[2]
P3 (OTMS)	TC/BG	SiO_2_ (300 nm)	Gate—n-doped Si, Source & Drain—Au	max: 3.95, avg: 3.29	10^6^–10^7^; 8–15	[3]
P3 (NTMS)	TC/BG	SiO_2_ (300 nm)	Gate—n-doped Si, Source & Drain—Au	max: 3.87, avg: 3.18	10^6^–10^7^; 8–15	[3]
P4 (OTMS)	TC/BG	SiO_2_ (300 nm)	Gate—n-doped Si, Source & Drain—Au	max: 7.37, avg: 6.93	10^6^–10^7^; 1–5	[3]
P4 (NTMS)	TC/BG	SiO_2_ (300 nm)	Gate—n-doped Si, Source & Drain—Au	max: 7.16, avg: 6.77	10^6^–10^7^; 1–5	[3]
PDTzTI (Off-center spin coating)	TG/BC	CYTOP	Gate—Al; Source & Drain—Au	max: 1.61, avg: 1.29	10^7–^10^8^; 24	[4]
PDTzTI (On-center spin coating)	TG/BC	CYTOP	Gate—Al; Source & Drain—Au	Max: 1.22, avg: 0.87	10^7^–10^8^; 35	[4,20]
P2 (NBA-TVT)_4_	TG/BC	PMMA	Gate—Al; Source & Drain—Cr/Au	max: 1.85, avg: 1.33 ± 0.46	log[I_on_/I_off_].: 3.3 ± 0.2; 32 ± 3	[10]
P3 (NBA-DTBT)_4_	TG/BC	PMMA	Gate—Al; Source & Drain—Cr/Au	max: 3.25, avg: 2.67 ± 0.42	log[I_on_/I_off_].: 3.4 ± 0.1; 29 ± 2	[10]
P4 (NBA-DTBTff)_4_	TG/BC	PMMA	Gate—Al; Source & Drain—Cr/Au	max: 4.46, avg: 3.73 ± 0.53	log[I_on_/I_off_].: 3.6 ± 0.4; 33 ± 3	[10]
BDPPV	TG/BC	CYTOP	Gate—Al; Source & Drain—Ti/Au	max: 1.1, avg: 0.84	≈10^6^; 51	[18]
P2N2F-4FBT	TG/BC	PMMA (~600 nm)	Gate—Al; Source & Drain—Au	max: 1.24, avg: 1.08	10^5^; ≈40	[21]
PBTI1 (Off-center Spin Coating)	TG/BC	CYTOP (~400 nm)	Gate—Al; Source & Drain—Au; CsF spin-cast onto Au S/D electrodes	max: 3.71, avg: 2.67	10^6^; 30	[29]
PBTI1 (On-center Spin Coating)	TG/BC	CYTOP (~400 nm)	Gate—Al; Source & Drain—Au; CsF spin-cast onto Au S/D electrodes	max: 1.53, avg: 1.19	10^6^; 25	[29]
PBTI2	TG/BC	CYTOP (~400 nm)	Gate—Al; Source & Drain—Au; CsF spin-cast onto Au S/D electrodes	max: 1.43, avg: 1.07	10^6^; 21	[29]
pPTT (OTMS)	TC/BG	SiO_2_ (300 nm)	Gate—n-doped Si, Source & Drain—Au	max: 2.11, avg: 1.87 ± 0.22	10^5^–10^6^; 3 ± 1	[31]
pSNT (OTMS)	TC/BG	SiO_2_ (300 nm)	Gate—n-doped Si, Source & Drain—Au	max: 4.87, avg: 3.96 ± 0.78	10^5^–10^6^; 8 ± 2	[31]
pSNT (NTMS)	TC/BG	SiO_2_ (300 nm)	Gate—n-doped Si, Source & Drain—Au	max: 5.35, avg: 4.58 ± 0.39	10^6^–10^7^; 1 ± 0.2	[31]
PBTI	TG/BC	CYTOP	Gate—Al; Source & Drain—Cr/Au	max: 2.60, avg: 2.50	10^6^; 20	[32]
P(BTI-BTI2)	TG/BC	CYTOP	Gate—Al; Source & Drain—Cr/Au	max: 1.23, avg: 0.82	10^6^; 17	[32]
PCNDFDE-DPP	TG/BC	CYTOP	Gate—Al; Source & Drain—Au	max: 1.07, avg: 0.98	10^3^; 21	[33]
P2 (Vacuum) (OTMS)	TC/BG	SiO_2_	Gate—n-doped Si, Source & Drain—Au	max: 2.55, avg: 2.18	10^6^–10^7^; −9 ± 5	[35]
P2 (Air) (OTMS)	TC/BG	SiO_2_	Gate—n-doped Si, Source & Drain—Au	max: 1.87, avg: 1.66	10^5^–10^6^; 5 ± 2	[35]
DPPTh-4ClTVT (PEIE) (spin coating)	TG/BC	PMMA (~600 nm)	Gate—Al; Source & Drain—Ti/Au	max: 1.29, avg: 1.06	~10^5^; 5.0–8.6	[34]
DPPTh-4ClTVT (PEIE) (bar coating)	TG/BC	PMMA (~600 nm)	Gate—Al; Source & Drain—Ti/Au	max: 1.44, avg: 1.18	~10^5^; 4.7–8.4	[34]

OTMS—octadecyltrimethoxysilane; NTMS—[3-(N,N-dimethylamino)propyl]trimethoxysilane; PEIE—polyethylenimine ethoxylated.

## Data Availability

No new data were created or analyzed in this study. Data sharing is not applicable to this article.
